# Combining PD-L1 blockade with a second-generation HSP110 inhibitor enhances antitumor immunity in colorectal cancer

**DOI:** 10.1038/s41419-026-08826-7

**Published:** 2026-05-19

**Authors:** María Jimena Abrey-Recalde, Flavie Mialhe, Thi Khanh Le, Abdelmnim Radoua, Lisa Lagorgette, Rim Belkaid, Adiilah Mamode Cassim, Daniel Gonzalez, Elise Jacquin, Jessica Gobbo, Frédéric Bibeau, Côme Lepage, Li Xu, Xingrui He, Valentin Derangère, Frédéric Lirussi, Gaëtan Jego, Xiang-Yang Ye, François Hermetet, Carmen Garrido

**Affiliations:** 1 Université Bourgogne Europe, INSERM, CTM UMR 1231, HSP-Pathies Team, équipe labellisée (Ligue Nationale Contre le Cancer), Dijon, France; 2 Université Bourgogne Europe, Fédération Francophone de Cancérologie Digestive (FFCD), INSERM, CTM UMR 1231, EPICAD Team, Dijon, France; 3 Université Bourgogne Europe, Centre de Lutte Contre le Cancer G.-F. Leclerc, Unicancer, INSERM, CTM UMR 1231, HSP-Pathies Team, équipe labellisée (Ligue Nationale Contre le Cancer), Dijon, France; 4 Université Marie et Louis Pasteur, CHU Besançon, Department of Pathology, Besançon, France; 5 Université Bourgogne Europe, CHU Dijon Bourgogne, Department of Digestive Oncology, Fédération Francophone de Cancérologie Digestive (FFCD), INSERM, CTM UMR 1231, EPICAD Team, Dijon, France; 6 School of Pharmacy, Hangzhou Normal University, Hangzhou, Zhejiang China; 7 Université Bourgogne Europe, Centre de Lutte Contre le Cancer G.-F. Leclerc, Unicancer, INSERM, CTM UMR 1231, TIRECs Team, Dijon, France; 8 Université Marie et Louis Pasteur, CHU Besançon, Department of Clinical Pharmacology and Toxicology, PACE Platform, Metabolomics Unit, Besançon, France; 9 Université Bourgogne Europe, INSERM, CTM UMR 1231, TIRECs Team, Dijon, France

**Keywords:** Drug development, Colorectal cancer, Cancer immunotherapy

## Abstract

In a previous work we demonstrated that pharmacological inhibition of wild type heat shock protein-110 (HSP110) could mimic an HSP110-inactive mutated phenotype reported to be associated with good prognosis in colorectal cancer (CRC) patients. We also demonstrated that HSP110 favored the formation of anti-inflammatory macrophages. We developed a second-generation HSP110 inhibitor called compound 7 (C7) through a hit-to-lead approach. Here, we deciphered its anti-cancer immune effect alone and associated with an anti-PD-L1/PD-1 targeting therapy. The study (FACS, IHC, immunoblots, qPCR, shRNA approaches) included syngeneic CRC mouse models (CT26/BALB/c and MC38/C57BL/6), 3D human CRC (HCT 116, HT-29) spheroids and primary human macrophages and lymphocytes isolated from buffy coats, and tumor biopsies from 134 CRC patients of the Prodige-13 trial. C7 specifically inhibited HSP110, leading to significant tumor growth suppression in both mice bearing CT26 and MC38 tumors and our human spheroids models incorporating immune cells. C7 reshaped the tumor microenvironment by promoting a pro-inflammatory phenotype. Studies in 2D and 3D co-cultures of human macrophages and lymphocytes indicated that C7’s effect involved a direct action on macrophages. C7 also induced the expression of the immune check point PD-L1 both in macrophages and tumor cells. Combining C7 and an anti-PD-L1 antibody resulted in a more effective tumor regression both in the immune check point inhibitor resistant CT26- and in the non-resistant MC38- tumor-bearing mouse model. Finally, we established a clinical relevance of HSP110 effect on macrophages by showing a correlation between HSP110 and the anti-inflammatory biomarker CD163 in a CRC patients’ cohort. These findings demonstrate that pharmacological inhibition of HSP110 alters pro-tumoral macrophages and can overcome resistance to immune check point inhibitors.

## Introduction

Tumor initiation and progression require the metabolic reprogramming of cancer cells, underscoring an intense need for chaperons such as the heat shock proteins (HSPs) for their survival. Targeting HSPs is, therefore, an appealing sensitization therapeutic strategy in cancer. Aside from one inhibitor targeting HSP27, all HSP inhibitors under clinical trials focus on HSP90. However, more than 20 years after their discovery, both natural and synthetic inhibitors typically suffer from limited efficacity [1, 2].

We and others demonstrated that HSP110 might be a better target. We reported in colorectal and gastric cancers microsatellite instable the presence of a deletion that inactivated HSP110. The expression of this mutation, which was produced at the detriment of wild type HSP110, correlated with an excellent patient’s outcome [3, 4].

Wild type HSP110 can be strongly expressed in different tumors cells such as colorectal cancer (CRC) and other cancers (epithelial, stomach, endometrium) [5]. Its expression is further induced by specific stress conditions provoked by the hostile tumor microenvironment and cancer treatments, such as chemotherapy or radiation [6]. Gene expression profiling in primary CRC has linked HSP110 to appearance of metastasis and poorer prognosis [7]. CRC cells with reduced HSP110 expression exhibit poor proliferation and are prone to apoptosis, which can be restored by re-expressing HSP110 in the cells [3, 8]. At the molecular level HSP110 have been shown to chaperone the transcription factor STAT3 [8] and the transcription repressor BCL6 [9].

HSP110 can be secreted and is abundant in the tumor microenvironment [8, 10, 11]. While the role of extracellular HSP110 remains largely unknown, our in vitro studies have shown that recombinant HSP110 promotes the differentiation of macrophages towards a tolerogenic phenotype [10]. Consistent with these findings, tumors expressing the mutant HSP110, compared to tumors expressing wild type HSP110, exhibit increased infiltration of immune cells [12–14] and a reduced number of anti-inflammatory tumor-associated macrophages (TAMs) [8]. Because only a small percentage of patients (around 15%) possess this HSP110 mutation associated with a therapeutic response [3], we hypothesized that the pharmacologic inhibition of HSP110 could mimic the HSP110-inactive phenotype in patients not bearing the mutation. Structural/modeling studies allowed us to select two small molecules, compounds 33 and 52, which inhibited HSP110 and induced tumor regression in CRC mouse models [15]. From hit-to-lead, we recently obtained a v2.0 molecule (C7) with a better solubility, stability, and specificity than the initial hits [16].

Herein, we deciphered the C7 anti-tumor immune effect mediated through pro-inflammatory macrophages in two CRC mice models (CT26 and MC38) and in human CRC spheroids, giving us a rational to explore a combinational therapy with immune check point inhibitors (ICIs) such as anti-PD-L1. The association of HSP110 with anti-inflammatory TAMs was also demonstrated in CRC patients’ resections. Overall, our findings highlight the potential of HSP110 inhibition as a valuable strategy to improve the efficacy of ICIs. This approach offers a promising avenue for future translation to patients who may not respond adequately to a PD-L1/PD-1 immunotherapy.

## Methods

### Colorectal cancer cell lines

CT26 (RRID:CVCL_7254), MC38 (RRID:CVCL_B288), HT-29 (RRID:CVCL_0320) and HCT 116 (RRID:CVCL_0291) CRC cell lines, purchased from the American Type Culture Collection (ATCC), were cultured at 37 °C in a humidified 5% CO_2_ atmosphere in Roswell Park Memorial Institute (RPMI)-1640 Medium (CT26) or in Dulbecco’s Modified Eagle’s Medium (DMEM) (MC38, HT-29, HCT 116) with 10% (v/v) fetal bovine serum (FBS) supplemented with 1% penicillin–streptomycin/amphotericin B (PSA) (Dutscher). Cells were regularly tested for mycoplasma contamination.

### Generation of CT26 HSP110 knockdown cells and HT-29 and HCT 116 mKate2-positive cells

MISSION® pLKO.1-puro non-target shRNA Control Plasmid (SHC016-1EA) and MISSION® pLKO.1-puro-CMV-tGFP hsph1 targeting shRNA plasmid (TRCN0000336031) were purchased from Sigma-Aldrich. Plasmid OM199 (pAIP_CMV_mKate2_IRES_PuroR) was kindly shared by Dr. Olivier Micheau (CTM-INSERM U1231). For lentivirus production, each plasmid was co-transfected with the packaging (psPAX2, RRID:Addgene_2260) and envelope (pCMV-VSV-G, RRID:Addgene_8454) vectors into HEK293T cells using jetPrime transfection reagent (Polyplus-transfection). Lentivirus was harvested at 48 h post-transfection from the supernatants, and mixed with 5 µg/mL polybrene (Selleckchem) to increase the infection efficiency. CT26 cancer cells were infected with the lentivirus and were selected by cell sorting (FACS ARIA III, BD Biosciences): cells expressing green fluorescent protein (GFP^+^) were isolated in 96-well plates to obtain clones. Similarly, HT-29 and HCT 116 cells were infected with OM199 lentivirus, and mKate2-positive cells were enriched by flow cytometry.

### Compounds

The first-generation HSP110 inhibitor called compound 33 and the second-generation compound 7 were synthesized by Synthenova laboratory (Hérouville-Saint-Clair, France) as already described [16]. Compounds C33 and C7 were dissolved in DMSO (Sigma-Aldrich) and stored at −20 °C. To achieve indicated concentrations, compounds were diluted in culture medium for in vitro assays or with corn oil for in vivo assays (Sigma-Aldrich). Anti-mouse PD-L1 (B7-H1) antibody was purchased from Bioxcell (#BX-BE0101, Clone 10 F.9G2) and stored at 4 °C. For in vivo assays, anti-PD-L1 monoclonal antibody was dissolved in sterile phosphate buffered saline (PBS). Controls with PBS or DMSO were carried out in each assay.

For co-culture assays, anti-human PD-1 (#BX-SIM0003, Clone Nivolumab) antibody was purchased from Bioxcell and stored at 4 °C.

### Human 3D spheroids

For tumor spheroid formation, HT-29 and HCT 116 mKate2-positive cells were washed, trypsinized, and resuspended in DMEM supplemented with 10% FBS. Cells (8000 per well) were seeded into Nunclon™ Sphera™ 96-well ultra-low attachment plates (ThermoFisher Scientific) at a volume of 100 µL per well, centrifuged at 300 g for 10 min at room temperature, and incubated for 48–72 h to allow spheroid formation. Peripheral blood mononuclear cells (PBMCs) were then added at effector-to-target (E:T) ratios of 1:1. C7 treatment was added in 50 µL per well to reach a final concentration of 40 µM. A vehicle control was prepared with an equivalent concentration of DMSO. Spheroid proliferation was monitored in real time using the Incucyte S3 Live-Cell Analysis System (Sartorius, France).

### Cultures of macrophages and lymphocytes from PBMCs

Buffy coats were obtained from the “Etablissement Français du Sang” (EFS, Dijon) and PBMC were isolated using Ficoll. After adhesion, monocytes were cultivated in RPMI 10% FBS with 100 ng/mL human GM-CSF or M-CSF (#130-093-866, #130-096-492, Miltenyi Biotec) for 6 days, then respectively activated with 20 ng/mL of IFNγ (#130-096-484, Miltenyi Biotec) and 100 ng/mL LPS (#L2887, Sigma-Aldrich) for an M1-like phenotype or 20 ng/mL of IL-4 (#130-093-922, Miltenyi Biotec) for an M2-like phenotype during 48 h. When indicated, the HSP110 inhibitors C33 or C7 were added during the activation.

In some experiments, the culture supernatant after monocyte adhesion was collected to recover T cells from the same donors. T lymphocytes were activated for 3 days in complete medium (RPMI supplemented with 10% FBS) containing TransAct Human CD3/CD28 (10 µL per 1 ×10⁶ cells/mL) and IL-2 (10 ng/mL). Cultures were refreshed every two days with complete medium supplemented with IL-2. Co-cultures of differentiated M2 type macrophages, treated or not for 48 h with 40 µM of C7, with activated T lymphocytes were carried out in the presence or absence of αPD-1 (20 µg/mL) for 72 h.

For the co-culture of the macrophages or/and T cells with tumor spheroids, differentiated macrophages were detached using Versene solution (ThermoFisher Scientific, France), washed, and resuspended in RPMI containing 10% FBS. 8000 macrophages were carefully added to each spheroid well to achieve a 2:1 cancer cell-to-macrophage ratio. T-cells were then added with the ratio 1:1. The proliferation of spheroids was then monitored in real-time using the Incucyte S3 Live-Cell Analysis System.

### Western blot analysis

For Western blot analysis, whole-cell lysates were subjected to SDS-PAGE and transferred into PVDF transfer membrane (Millipore) for analysis. The following primary antibodies were used: anti-HSP110 (RRID:AB_2119247), 1:1000), anti-HSC70 (RRID:AB_627761, 1:1000) and anti-GAPDH (RRID:AB_627678, 1:1000) were purchased from Santa Cruz Biotechnology, anti-HSP90α (RRID:AB_2120934, 1:1000) was purchased from Thermo Fisher Scientific, anti-HSP70 (RRID:AB_10013742, 1:1000) was purchased from Enzo Life Sciences, and anti-PD-L1 (RRID:AB_2773715, 1:1000) was purchased from Abcam. HRP conjugated anti-mouse IgG (RRID:AB_330924, 1:5000) and HRP-conjugated anti-rabbit IgG (RRID:AB_2099233, 1:5000) secondary antibodies were purchased from Cell Signaling Technology.

### In vivo studies

All experiments with mice were performed in compliance with preclinical ethical guidelines and approved by the local ethical committee for animal welfare of the Université Bourgogne Europe (C2EA Grand Campus, Dijon, France) and the French Ministry of Higher Education, Research and Innovation (under reference APAFIS#25224). 8–10-week-old female immunocompetent BALB/c (RRID:MGI:2161072) or C57BL/6 mice (RRID:MGI:2159769) were purchased from Charles River Laboratories (Ecully, France).

CT26 WT/sh110 cells (5 × 10^5^) or MC38 cells (5 × 10^5^) were resuspended in 100 µL sterile PBS and injected subcutaneously into the right flank of BALB/c or C57BL/6 female mice, respectively (Charles River Laboratories) using a 27 G needle. One week later, tumors were measured using a caliper and mice were randomized before treatments. Tumor volumes and body weights were measured three times a week. Tumor size was determined by measuring the length and width of the tumors using the formula length × width^2^/2.

For drug treatment, mice were intraperitoneally treated with a control vehicle, C33 or C7 at 5 mg/kg, αPD-L1 antibody at 200 µg/mouse or the combination three times a week. Mice were euthanized when tumors reached 1500 mm^3^ and tumors were collected for flow cytometry and immunofluorescence analysis.

To evaluate the safety profile of compound C7, we conducted a 6-week in vivo study in BALB/c mice. Animals received intraperitoneal injections of C7 at doses of 5 or 10 mg/kg, three times per week. Throughout the treatment period, general condition and body weight were monitored twice weekly according to ICH regulatory guidelines M3 (R2). At the end of the study, major organs (liver, kidney, spleen, lung, and heart) were collected for gross examination and weighing. Organs were then fixed, sectioned, and stained with hematoxylin and eosin for histopathological analysis. Slides were digitalized using a Nanozoomer HT2.0 (Hamamatsu Photonics, Japan) at 20X magnification to produce whole slide imaging files. Peripheral blood was also collected post-mortem and analyzed using an automated hematology analyzer (SCIL Vet ABC+, Oostelbeers, the Netherlands) to perform complete blood counts.

### Immunofluorescence

Toxicity was evaluated by Cleaved Caspase-3 staining (C3C) in intestinal crypts of tumor-bearing control mice and either treated with compound C7 or C33. Dissected tumors and intestinal crypts were embedded in paraformaldehyde (PFA) during 24 h, then embedded in paraffin and tissue sections were dried onto microscope slides. For immuno-staining, tissue sections were rehydrated, then samples were incubated overnight at 4 °C with the primary anti-phospho-STAT3 antibody (RRID:AB_2491009, 1:200, Cell Signaling), and anti-C3C (RRID:AB_2341188, 1:200, Cell Signaling) in blocking solution PBS with 2% bovine serum albumin and 0.1% Triton X-100. Primary antibodies were detected using Alexa Fluor 488-coupled secondary antibody (RRID:AB_2633280, 1:500, Thermo Fisher Scientific). Nuclear labeling was performed with ProLong™ Gold Antifade Mountant with DAPI (#P36931, Thermo Fisher Scientific). Pictures were taken in random fields for each labeling, and analyzed using ZEN Microscopy Software (ZEISS).

### Flow cytometry analysis

Tumors from mice were collected and digested with Mouse Tumor Dissociation kit and the gentleMACS™ Dissociator according to the manufacturer’s protocol (#130-096-730 and #130-093-235, Miltenyi Biotec). For cytokines analysis, cells were incubated for 4 h with Cell Stimulation Cocktail containing phorbol 12-myristate 13-acetate and ionomycin (#00-4970-03, Thermo Fisher Scientific). Then, cells were counted and directly stained with Fixable Viability Stain 780 (1:5000, RRID:AB_2869673, BD Biosciences). Cells were stained with membrane antibodies, then permeabilized and fixed with the Cytofix/Cytoperm™ Solution kit according to the manufacturer’s recommendations (#554714, BD Biosciences). Finally, intracellular staining was performed to assess cytokine expression. Cells were analyzed with a Cytek® Aurora (Cytek Biosciences), followed by FlowJo software v.10 (BD Biosciences) analysis. Fluorochrome-conjugated antibodies specific of mouse TAMs, tumor infiltrating lymphocytes (TILs), myeloid-derived suppressor cells (MDSCs), and dendritic cells (DCs) were purchased from Bio Legend (BL), BD Biosciences (BD), Miltenyi Biotec (MB) or Thermo Fisher Scientific (TF) and listed above: mouse CD45 (RRID:AB_2651134, 1:600, BD), mouse F4/80 (RRID:AB_893478, 1:200, BL), mouse CD80 (RRID:AB_1645212, 1:200, BD), mouse CD206 (RRID:AB_2538348, 1:100, TF), mouse PD-L1 (RRID:AB_2073556, 1:100, BL), mouse CD4 (RRID:AB_2819537, 1:600, MB), mouse CD8 (RRID:AB_2564385, 1:600, BL), mouse PD-1 (RRID:AB_2159183, 1:200, BL), mouse TIM-3 (RRID:AB_2814028, 1:200, BL), mouse LAG-3 (RRID:AB_2133343, 1:200, BL), mouse IFNγ (RRID:AB_2734493, 1:200, BL), mouse IL-2 (RRID:AB_315300, 1:200, BL), mouse Granzyme B (GzmB; RRID:AB_2728383, 1:200, BL), mouse CD11b (RRID:AB_2871246, 1:200, BD), mouse CD11c (RRID:AB_1727421, 1:100, BD), mouse CD103 (RRID:AB_2629724, 1:100, BL), mouse Ly-6G (RRID:AB_2565881, 1:200, BL), and mouse Ly-6C (RRID:AB_2737736, 1:200, BD).

For the in vitro culture studies, after the isolation of PBMCs and the differentiation towards M1-like of M2-like macrophages, cells were activated and treated or not with compound C33 or C7 at 40 µM during 48 h. Macrophages were washed with PBS and detached with accutase (#A6964, Sigma-Aldrich) before staining. Cells were stained with Fixable Viability Stain 780 (1:5000, RRID:AB_2869673, BD Biosciences). Then, fluorochrome-conjugated antibodies specific of human markers were purchased from Bio Legend (BL) or BD Biosciences (BD) and listed above: human CD45 (RRID:AB_2869519, 1:200, BD), human CD11b (RRID:AB_396939, 1:200, BD), human CD80 (RRID:AB_2076147, 1:200, BL), human HLA-DR (RRID:AB_10563765, 1:200, BD), human CD206 (RRID:AB_394065, 1:200, BD), human Dectin-1 (RRID:AB_2738819, 1:200, BD), human PD-L1 (RRID:AB_2629582, 1:200, BL), human CD8 (RRID:AB_2871326, 1:200, BD), and human IFNγ (RRID:AB_2801101, 1:200, BL).

For co-culture assays, supernatants containing activated T cells from the same donors were collected for co-culture with differentiated M2-like macrophages. These cocultures were conducted in the presence or absence of anti-PD-1 antibody. T cells were then collected from the supernatant and stained with specific membrane markers and cytokines. CD8^+^ T cells were labeled with CFSE staining (#C34554, Thermo Fisher Scientific) according to the manufacturer’s protocol.

### Patients and study design

The study included a cohort of 68 stage II and 66 stage III CRC patients issued from the PRODIGE-13 trial (2009 patients included;NCT00995202) [17], a randomized, prospective, multicenter controlled phase III trial evaluating by factorial plan the impact of i) intensive radiological monitoring (alternating abdominal ultrasound (US)/CTscan/3 m) versus standard monitoring (US/3 m and thoracic radiography/6 m) and ii) CarcinoEmbryonic Antigen (CEA) assessment versus no assessment, in the follow-up of resected stage II or III CRC with no evidence of residual disease on baseline post-surgical investigation in France and Belgium.

All clinical data and tissue specimens were used in compliance with the Fédération Francophone de Cancérologie Digestive (FFCD) guidelines. Approval for the study and patient consent were obtained from the FFCD with the reference numbers EudraCT 2009-A00536-51 and CPP 2009/34, respectively. Patients’ clinical characteristics are presented in Table 1.

**Table 1:** Clinical characteristics of the PRODIGE-13 cohort patients included in this study.

Item	*N* (total) (134)	Stage II (68)	Stage III (66)	*P*-value
Sex				0.962
Male	85 (63.4%)	43 (63.2%)	42 (63.6%)	
Female	49 (36.6%)	25 (36.8%)	24 (36.4%)	
Location of tumor				0.630
Right colon	59 (44.0%)	32 (47.1%)	27 (40.9%)	
Left colon	58 (43.3%)	29 (42.6%)	29 (43.9%)	
Rectum	17 (12.7%)	7 (10.3%)	10 (15.2%)	
Number of lymph nodes				<0.0001
N0	69 (51.5%)	68 (100%)	1 (1.5%)	
N1	44 (32.8%)	0 (0%)	44 (66.6%)	
N2	21 (15.7%)	0 (0%)	21 (31.8%)	
MSS/MSI status				<0.0001
MSS	63 (47.0%)	52 (76.5%)	11 (16.7%)	
MSI	14 (10.5%)	13 (19.2%)	1 (1.5%)	
Non-evaluable	55 (41.0%)	1 (1.5%)	54 (81.8%)	
NA	2 (1.5%)	2 (2.9%)	0 (0%)	
Tumor size				0.030
T1	2 (1.5%)	0 (0%)	2 (3.0%)	
T2	14 (10.4%)	7 (10.3%)	7 (10.6%)	
T3	100 (74.6%)	56 (82.4%)	44 (66.7%)	
T4	17 (12.7%)	4 (5.9%)	13 (19.7%)	
NA	1 (0.7%)	1 (1.5%)	0 (0%)	
Grade				0.595
Well/Moderately	125 (93.3%) (93.3(93.3%)	64 (94.2%)	61 (92.4%)	
Poorly or undifferenced	4 (3.0%)	1 (1.5%)	3 (4.5%)	
NA	5 (3.7%)	3 (4.4%)	2 (3.0%)	
Treatment				<0.0001
Chemotherapy	70 (52.2%)	4 (5.9%)	66 (100%)	
No chemotherapy	64 (47.8%)	64 (94.1%)	0 (0%)	

*N* number of patients, *MSS* microsatellite stable, *MSI* microsatellite instable, *NA* not available.

### HSP110 and CD163 staining and analysis from patients’ biopsies

Histological slides staining was carried out using Autostainer 48 (Agilent), anti-HSP110 (RRID:AB_10858730) and anti-CD163 [EPR19518] (RRID:AB_2753196) primary antibodies from Abcam. After counterstaining and mounting, slides were digitalized using a Nanozoomer HT2.0 (Hamamatsu Photonics, Japan) at 20X magnification to produce whole slide imaging files. Quantification profiles of the digitized whole slide images were done using QuPath software (V2) under bright field fast cell counting and with positive cell and nucleus detection settings. A mean of 33 different areas of tumor cells, representing at least 0.8 mm^2^ for each slide, was analyzed for HSP110 and CD163 staining. A cut-off for each subset was determined on diaminobenzidine intensity (brown staining) and automatically applied on every cell detected in annotated areas (i.e. negative or low (1+), middle (2+), strong (3+) HSP110 staining; negative or positive CD163 staining). The HSP110 H-Score was then calculated with the following formula: H-score = [1× (% cells 1+) + 2× (% cells 2+) + 3× (% cells 3+)] [18].

### Statistical analysis

All statistical analyses were performed using GraphPad Prism v8.3.0 (GraphPad Software Inc.). No statistical methods were used to predetermine sample size or ensure adequate power for a predefined effect size. To compare differences between two groups, unpaired or paired *t*-tests were employed. The normality of continuous variables was assessed. For non-normally distributed data, the Mann–Whitney test and Wilcoxon test were applied. One-way ANOVA or Kruskal–Wallis tests were used for the comparison of multiple groups of different sizes or more groups within a single condition, respectively, depending on the normality. Two-way ANOVA with Sidak’s or Tukey’s multiple testing correction was used for the tumor growth analysis. Exact *p*-values are indicated in the figure legends. Quantitative variables of the PRODIGE-13 cohort were expressed as median and interquartile range (IQR) and compared using the Mann–Whitney test. Categorical variables were expressed as count and percentage and compared using the Chi square test. Boxplots were drawn with median, quartiles, and Tukey’s whiskers. *P*-values < 0.05 were considered significant.

## Results

### Pharmacological inhibition of HSP110 increases the pro-inflammatory tumor environment

**Fig. 1:**
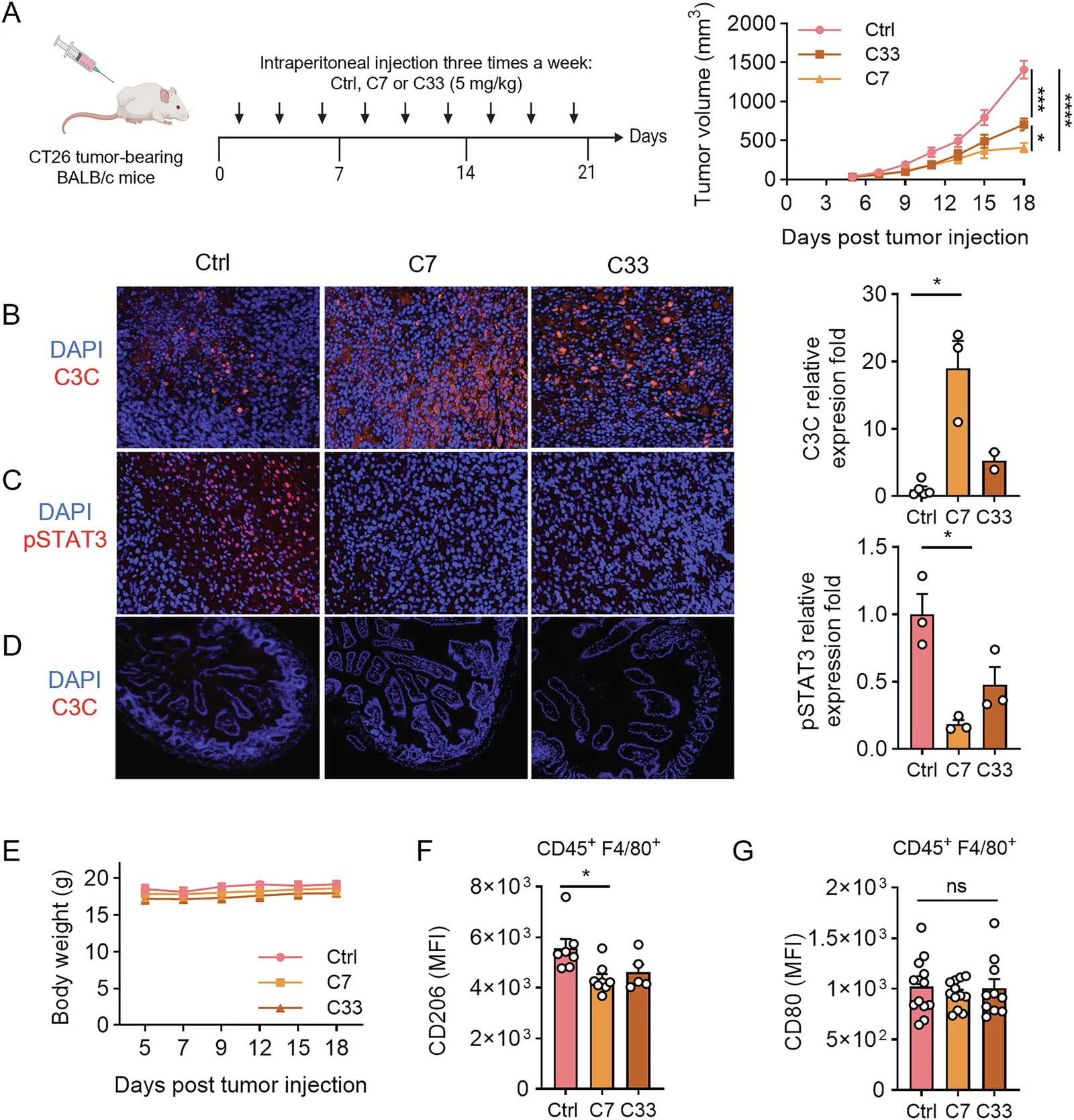
First-in-class HSP110 inhibitors (C7, C33) induce tumor volume reduction in mice. **A** Tumor volume monitoring of CT26 cells in BALB/c mice treated with C7 or C33 (5 mg/kg, i.p., three times per week) or vehicle (control, Ctrl). Mean tumor volume (±SEM) is represented (*n* = 10) Two-way ANOVA. *p < 0.05; ***p < 0.001; ****p < 0.0001. **B** Tumor cell apoptosis and **C** STAT3 phosphorylation were studied by immunofluorescence labeling cleaved caspase-3 (C3C) or pSTAT3, respectively, on dissected tumors from mice. Normalized fluorescence over DAPI of pSTAT3 or C3C is represented (*n* = 3). Kruskal–Wallis test; *p < 0.05. One representative image is shown. Scale bar = 50 µm. **D** Absence of toxicity associated to pharmacological inhibition of HSP110 was evaluated by C3C staining in intestinal crypts of tumor-bearing control mice and either treated with compound C7 or C33. **E** Animal weight was evaluated in control-, C7- or C33-treated mice (*n* = 5). **F** Assessment of CD206 expression (indicative of M2-like macrophages) or **G** CD80 expression (indicative of M1-like macrophages) in TAMs from control mice and C7 or C33-treated. Data are depicted as means ± SEM. One-way ANOVA. *p < 0.05; ns non-significant.

We previously demonstrated the antitumoral effect of a first-generation inhibitor of HSP110, the small molecule C33 [15]. More recently, a chemical modification of the central bipyridyl scaffold allowed the selection of a more soluble and bioavailable C33-derivative, named C7 [16]. In this work, we first compared the antitumor effects of C7 and C33 in BALB/c mice bearing colorectal carcinoma CT26 tumors. Mice with subcutaneous CT26 tumors measuring 30 mm^3^ were treated with the vehicle (DMSO), C7, or C33 at a dose of 5 mg/kg administered intraperitoneally three times a week during 21 days. Mice treated with C33 showed a 50% reduction in tumor growth compared to control mice. This reduction in tumor growth was more pronounced (75–80%) after treatment with C7 (Fig. 1A). We confirmed this observation by analyzing tumor cell apoptosis, assessed by cleaved caspase-3 staining in the tumor slides, showing a higher number of apoptotic cancer cells in the animals treated with C7 compared to C33 (Fig. 1B). These findings align with the observed reduction in the levels of phosphorylated STAT3 on tumor slides (Fig. 1C), a known effect of HSP110 that contributes to cell survival [8]. It is worth mentioning that, at the doses used, no toxicity was detected with both of the compounds, determined by the absence of apoptosis in epithelial cells from the intestinal crypts (Fig. 1D) and the lack of loss of body-weight (Fig. 1E).

To assess a longer-term safety of compound C7, we conducted a 6-week in vivo study in mice administered C7 intraperitoneally at 5 mg/kg (efficiency dose used in Fig. 1) or at the higher dose of 10 mg/kg, three times per week. The general condition and body weight of the mice were monitored every 2 days and showed no evidence of toxicity or weight loss (Supplementary Fig. S1A). At the study endpoint, macroscopic examination and weighing of major organs (lung, heart, spleen, kidney, and liver) (Fig. S1B) revealed no signs of tissue damage and abnormalities (e.g., necrosis, hemorrhage, splenomegaly). Histopathological assessment of hematoxylin and eosin–stained sections showed no treatment-related microscopic alterations or lesions in any of the examined tissues (Supplementary Fig. S1C). In addition, complete blood counts showed no hematologic abnormalities (Supplementary Fig. S1D). These findings indicate that this prolonged C7 administration was not associated with organ-specific or systemic toxicity, even at 10 mg/kg, a dose twice as high as the effective dose required for tumor growth reduction.

To demonstrate that the anti-tumor effect of C7 was through HSP110 inhibition, we conducted experiments in mice bearing CT26 tumor cells HSP110 knockdown. We chose a knockdown strategy instead of knockout because the latter can trigger a compensatory upregulation of other HSPs. Two clones of CT26 shHSP110 cells were selected, in which HSP110 was strongly depleted while the expression levels of other related HSPs, such as HSP70 and HSP90, remained unaffected (Supplementary Fig. S2A). We implanted CT26 shRNA control or shHSP110 tumor cells subcutaneously in BALB/c mice. Subsequently, we treated these mice with either vehicle or C7, as described above. shHSP110 CT26 tumors were significantly smaller than CT26 shRNA control tumors, confirming the well-reported role of HSP110 in tumor cell growth. Treatment with C7, while decreasing the size of the shRNA control CT26 tumors (expressing HSP110), it did not have any significant impact on the shHSP110 tumors (Supplementary Fig. S2B), supporting the notion that the antitumoral properties of compound C7 are linked to its target HSP110.

The anticancer effect of C7 was confirmed on a human 3D tumor spheroid model. Fluorescent human colorectal HCT 116 or HT-29 spheroids (mKate2-positive) were generated in ultra-low-attachment plates. Spheroids were subsequently incubated with PBMCs from healthy donors, in the presence or absence of C7 (40 µM) (Supplementary Fig. S3A). Real-time imaging (IncuCyte S3) was performed during 96 h, and fluorescence intensity was normalized to baseline values. C7 treatment reduced spheroid growth compared with control conditions (Supplementary Fig. S3B, C), consistent with the results obtained in our mice models.

Previously, we reported that HSP110 overexpression by tumor cells affected macrophage polarization and promoted a pro-tumor and anti-inflammatory M2-like phenotype [8, 10]. Thus, we investigated the phenotype of TAMs in the tumors from mice treated either with a vehicle or C7. Flow cytometry analyses revealed that inhibition of HSP110 with C7 reduced the expression of pro-tumoral M2-like TAM markers like CD206 (Fig. 1F), without affecting the expression of M1-like macrophages markers, such as CD80 (Fig. 1G). Albeit non-significant, C33 treatment also induced a decrease in CD206 (Fig. 1F). Interestingly, we also observed that treatment with C7 increased the expression of PD-L1 in TAMs and tumor cells, an immune checkpoint associated with tumor adaptive immune resistance [19] (Supplementary Fig. S4A–C). We next evaluated the effect of C7 on other immune cells within the tumor bed. Although the inhibition of HSP110 by C7 did not have an impact on the number of MDSCs or DCs infiltrating the tumor (not shown), we observed an increase in PD-L1 expression in those cells (Supplementary Fig. S4D, E).

Altogether, these observations provide evidence of the superior anti-tumor effect of the second-generation compound C7 and of its ability to promote macrophages with a more pro-inflammatory phenotype. It also settles the rational for a combinational therapy associating an anti-PD-L1.

### Potentiation effect of C7 and anti-PD-L1 combinational therapy on tumor growth and TAMs

**Fig. 2:**
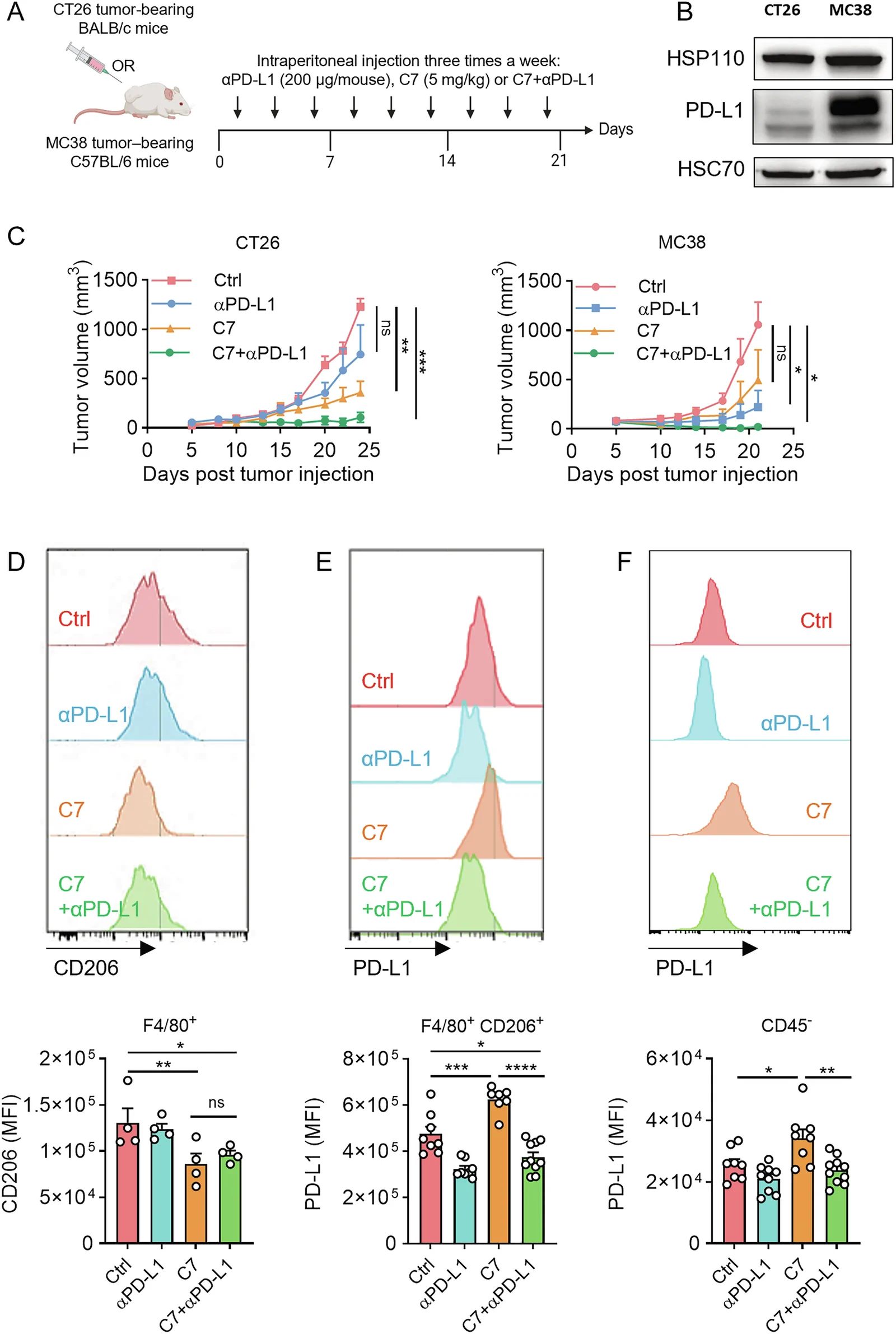
Pharmacological inhibition of HSP110 synergizes with anti-PD-L1 in vivo. **A** Schema depicting the treatment schedule. **B** PD-L1 and HSP110 protein expression in CT26 and MC38 cells assessed by Western blot. HSC70 is used as a loading control. **C** CT26 or MC38 tumor growth in mice control, treated with C7 (5 mg/kg), anti-PD-L1 (200 µg/mouse), or the combination of both. Treatment was administered intraperitoneally (i.p.) 3 times per week. Mean volume (± SEM) is displayed; 6 animals per group; Two-way ANOVA. *p < 0.05; **p < 0.01; ***p < 0.001; ns, non-significant (one representative experiment out of 3 is shown). **D** Measurement of CD206 expression by TAMs from control and treated mice using flow cytometry. Shown is the mean ± SEM (*n* = 7) of CD206 fluorescence intensity in F4/80^+^ cells. **E** PD-L1 expression by M2-like macrophages from tumors of control, C7 and/or anti-PD-L1 treated mice. Mean ± SEM (*n* = 8) of PD-L1 fluorescence intensity in M2-like macrophages (F4/80^+^CD206^+^) is depicted. **F** Expression of PD-L1 by tumor cells from control or treated mice. Mean ± SEM (*n* = 8) of PD-L1 fluorescence intensity in CD45^-^ cells is represented. One-way ANOVA: *p < 0.05; **p < 0.01; ***p < 0.001; ****p < 0.0001; ns non-significant.

We then studied the effect of C7 combined with anti-PD-L1 in the CRC mice CT26 and MC38 models, as depicted in Fig. 2A. The first model is described as resistant to PD-1/PD-L1 therapies. In contrast, the second is sensitive [20], which may be explained by the lower levels of PD-L1 expression exhibited by the CT26 cells compared to the MC38 cell line (Fig. 2B). We implanted CT26 and MC38 cells subcutaneously in BALB/c or C57BL/6 mice, respectively. When the size of the tumors reached a size of 30 mm^3^, animals were treated with anti-PD-L1, C7, or a combination of both (Fig. 2A). While treatment with anti-PD-L1 had no significant effect in the CT26 model but reduced tumor growth of the MC38 tumors as expected, treatment with C7 or in combination with anti-PD-L1 significantly reduced the tumor growth in both models (Fig. 2C, left and right panels, respectively). In all animals treated with the combined therapy, there was a more significant tumor regression compared to the monotherapies (Fig. 2C).

A potentiation of the therapeutic effect of anti-PD-L1 was also observed when associated with the HSP110 inhibitor C33. However, in line with its lower efficiency compared to C7, none of the animals experienced complete tumor regression (Supplementary Fig. S5A, B).

Next, we assessed the impact of the combined anti-PD-L1 + HSP110 targeted therapy on the phenotype of TAMs and their PD-L1 expression. For these experiments, animals were sacrificed one week after the beginning of the treatment. As expected, C7 alone or associated with anti-PD-L1 decreased the expression of the M2-like anti-inflammatory marker CD206 compared with non-treated or anti-PD-L1 alone conditions (Fig. 2D). The expression of PD-L1 was increased by C7 in tumor cells and M2-like TAMs. However, this expression was significantly reduced by anti-PD-L1 (Fig. 2E, F). Similar observations were made with C33 when combined with anti-PD-L1 (Supplementary Fig. S5B, C).

These findings strongly suggest that the immunomodulatory effect induced by HSP110 inhibition creates a suitable pro-inflammatory tumor microenvironment, which potentiates the effect of anti-PD-L1 in the both immune-responsive (MC38) and non-responsive (CT26) models.

### Combined C7 and anti-PD-L1 therapy in mice induces functional CD8^+^ TILS

**Fig. 3:**
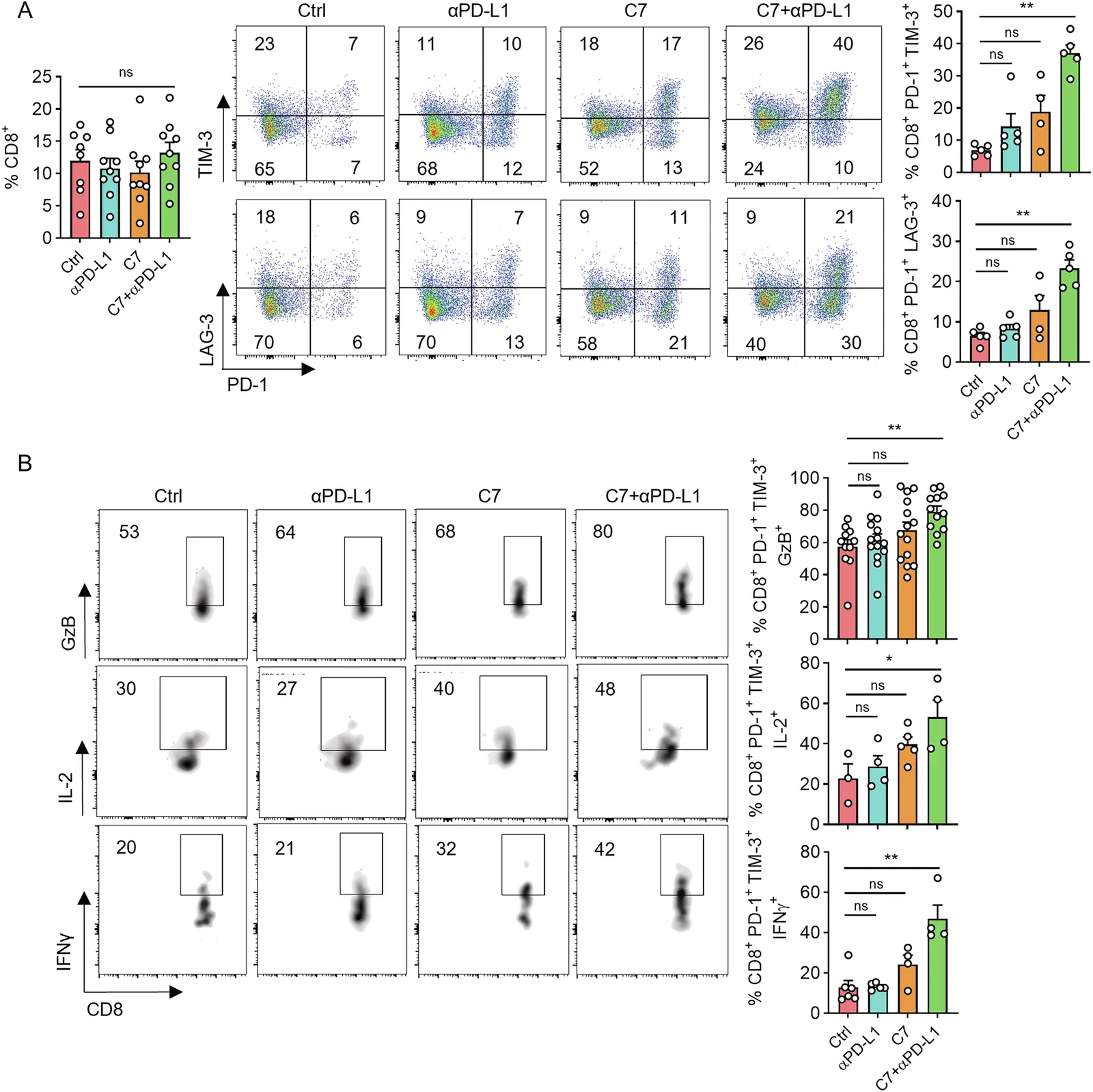
C7 and anti-PD-L1 synergistically induce functional CD8 + TILs in the tumor microenvironment. **A** Expression of the immune checkpoints PD-1, TIM-3 and LAG-3 in the membrane of tumor infiltrated CD8^+^ T cells from control and treated mice was analyzed by flow cytometry. Mean (± SEM, *n* ≥ 5) of percentage of CD8^+^ T cells co-expressing TIM-3 and PD-1 or LAG-3 and PD-1 is depicted. **B** Cytokine production by tumor infiltered CD8^+^ PD-1^+^ TIM-3^+^ T cells from control or treated mice evaluated by intracellular flow cytometry. Mean (± SEM, *n* ≥ 5) of percentage of CD8^+^ PD-1^+^ TIM-3^+^ T cells expressing granzyme B (GzB), interferon gamma (IFNγ) and interleukine-2 (IL-2) is represented. One-way ANOVA and Kruskal–Wallis test: *p < 0.05; **p < 0.01; ns non-significant.

To address whether treatment with C7 and anti-PD-L1 could restore ICI-induced cytotoxic T-cell responses in the CT26 tumor bed, we isolated TILs from the different groups of treated CT26 tumor-bearing mice and evaluated T-cell functionality. We analyzed the percentage of CD8 T-cells expressing the immune checkpoint markers PD-1, LAG-3, and TIM-3 and the intracellular expression of IFNγ, GzmB, and IL-2. While treatment with anti-PD-L1 or C7 alone did not induce a significant increase in the number of CD8^+^ T-cells co-expressing PD-1 and TIM-3 or PD-1 and LAG-3, an increase statistically significant was observed with the combination C7 + anti-PD-L1 (Fig. 3A). Interestingly, even though the expression of these markers is frequently associated with an exhausted state, those CD8^+^ T cells revealed an effector-like phenotype. Indeed, they significantly expressed higher levels of Grz B, IFNγ, and IL-2 compared with CD8^+^ T cells from tumors of control mice or treated with the monotherapies (Fig. 3B). These data suggest that while C7 treatment induces PD-L1 expression and a slight increase in the frequency of potentially exhausted CD8^+^ TILS, the addition of anti-PD-L1 can prevent CD8^+^ T cells dysfunction and ensure a sustained antitumor response.

### Pharmacological inhibition of HSP110 in vitro induces human pro-inflammatory macrophages, promoting T-cell proliferation

**Fig. 4:**
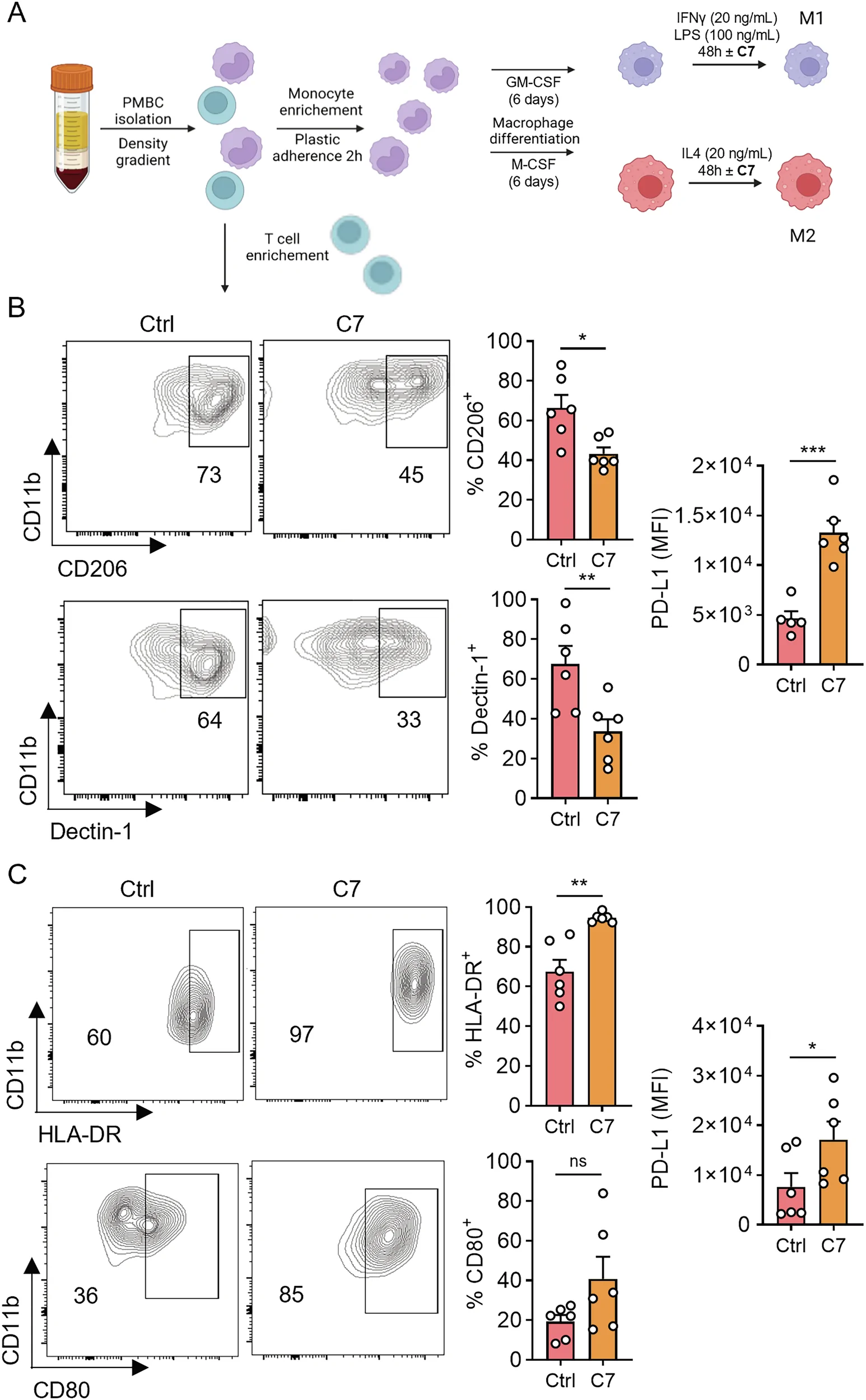
C7 induces macrophage polarization towards an M1 phenotype. **A** Schema depicting the isolation of PBMCs and macrophage differentiation protocol used. **B** Differentiated M2-like or **C** M1-like macrophages in the presence or not of C7 (40 µM) were assessed for the expression of CD206, Dectin-1, HLA-DR, CD80, and PD-L1 by flow cytometry. Results are presented as mean (± SEM, *n* = 6) of the percentage of positive cells or mean fluorescence intensity (MFI), respectively. Paired *t*-test and Wilcoxon test: *p < 0.05; **p < 0.01; ***p < 0.001; ns non-significant.

We next investigated if C7 could directly skew macrophages toward an inflammatory phenotype. We performed in vitro experiments with human monocytes isolated from buffy coats of healthy donors. Monocytes were polarized towards M1- or M2-like phenotypes and then, incubated with C7 or vehicle previous activation with IL-4 (M2-like) or LPS and IFNγ (M1-like), as depicted in Fig. 4A. 48 h after activation, their phenotype was evaluated. We observed that C7 induced a marked polarization toward a pro-inflammatory M1-like phenotype, evidenced by the downregulation of CD206 and Dectin-1 (Fig. 4B) and the upregulation of CD80 and HLA-DR markers (Fig. 4C). This effect of HSP110 inhibition with C7 in M2-like and M1-like macrophages was confirmed using the C33 molecule (Supplementary Fig. S6A, B). Interestingly, as observed in mice, PD-L1 expression was also induced by C7 treatment in M2- and M1-like macrophages (Fig. 4B, C, right panels).

**Fig. 5:**
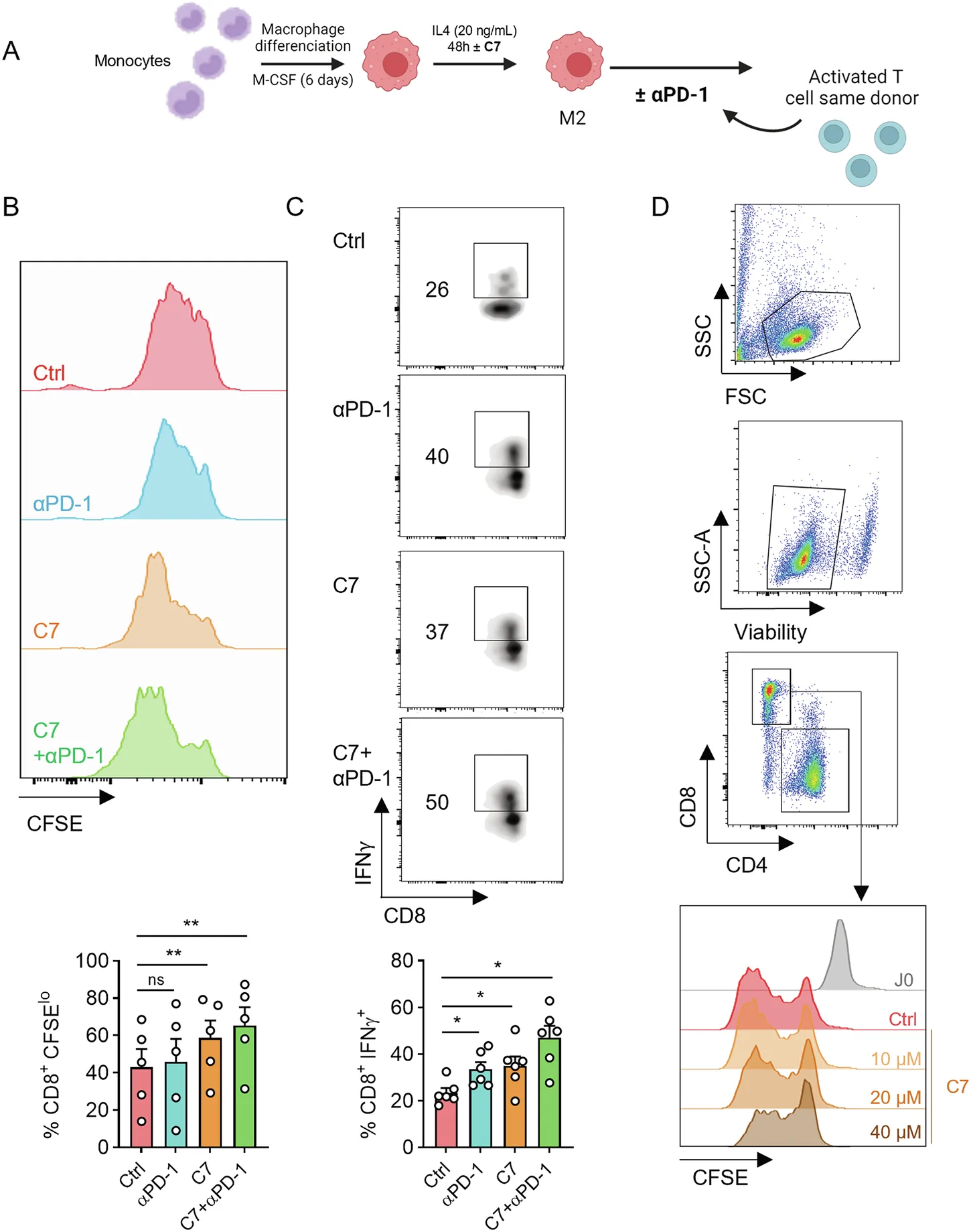
Combinatory effect of compound C7 and anti-PD-1 in vitro. **A** Schematic representation illustrating the coculturing of differentiated M2-like macrophages (which were either pre-treated with compound C7 (40 µM) or left untreated) with activated T cells from the same donors, in the presence or absence of αPD-1 (20 µg/mL). **B** Assessment of T cell proliferation through CFSE staining. Results are presented as mean (± SEM) of the percentage of CD8^+^ T cells with low CFSE staining (*n* = 5). **C** The production of IFNγ by CD8^+^ T cells was evaluated using flow cytometry, and the results are presented as the mean (± SEM, *n* = 5) of the percentage of CD8^+^ T cells positive for IFNγ staining. One-way ANOVA: *p < 0.05; **p < 0.01; ns non-significant. **D** T cell proliferation assessed by CFSE staining in activated T cells without macrophages and treated with increasing concentrations of C7 (*n* = 3).

To explore the hypothesis that HSP110 inhibition could activate T-cells indirectly through its effect polarizing macrophages towards a pro-inflammatory M1-like phenotype, we performed co-culture experiments with human macrophages and T lymphocytes isolated from the same buffy coats. Macrophages were polarized, preincubated with C7, and activated as described in Fig. 5A. After 48 h of activation, they were co-cultured with previously stimulated anti-CD3 and anti-CD28 T lymphocytes for 3 days. Then, the functionality of CD8^+^ T lymphocytes was studied by analyzing proliferation and IFNγ production by flow cytometry. As shown in Fig. 5B, C, CD8^+^ T lymphocytes co-cultured with M2-macrophages in the presence of C7 exhibited higher proliferation rates and increased production of IFNγ compared with the control co-cultures. Further, the addition anti-PD-1 in the co-culture augmented both processes. It is worth mentioning that C7 had no direct effect in primary human lymphocytes isolated from buffy coats (Fig. 5D).

Altogether, these results confirm that inhibiting HSP110 causes a shift in the macrophages’ phenotype towards a pro-inflammatory state. This shift is associated with and leads to the activation and expansion of CD8^+^ T lymphocytes. Furthermore, blocking the PD-L1 axis amplifies this effect.

We also assessed how HSP110 inhibition influences macrophage differentiation and the tumor-T cell cross-talk in the human 3D human CRC spheroid model. Fluorescent HT-29 spheroids (mKate2-positive) were incubated with macrophages derived from PBMCs that had been previously treated to induce a polarization toward an M1-like or an M2-like phenotype, in the presence or absence of C7 (40 µM for 48 h), and then co-cultured with or without autologous activated T cells (Supplementary Fig. S7A). Spheroid fluorescence was continuously monitored using the IncuCyte S3 system and normalized to baseline values. C7-conditioned M1-like macrophages reduced spheroid growth relative to controls, and this inhibitory effect was further potentiated in the presence of T cells (Supplementary Fig. S7B). Interestingly, when starting from M2-like macrophages, a comparable decrease in red fluorescence intensity was observed, indicating a functional repolarization of the macrophages toward a pro-inflammatory state that can enhance macrophage-T cell cooperation (Supplementary Fig. S7C). Together, these results demonstrate that C7-treated macrophages acquire antitumor activity in a human 3D spheroid model that recapitulates key immune interactions observed in vivo.

### Association between HSP110 and the number of anti-inflammatory macrophages in CRC patients’ biopsies

**Fig. 6:**
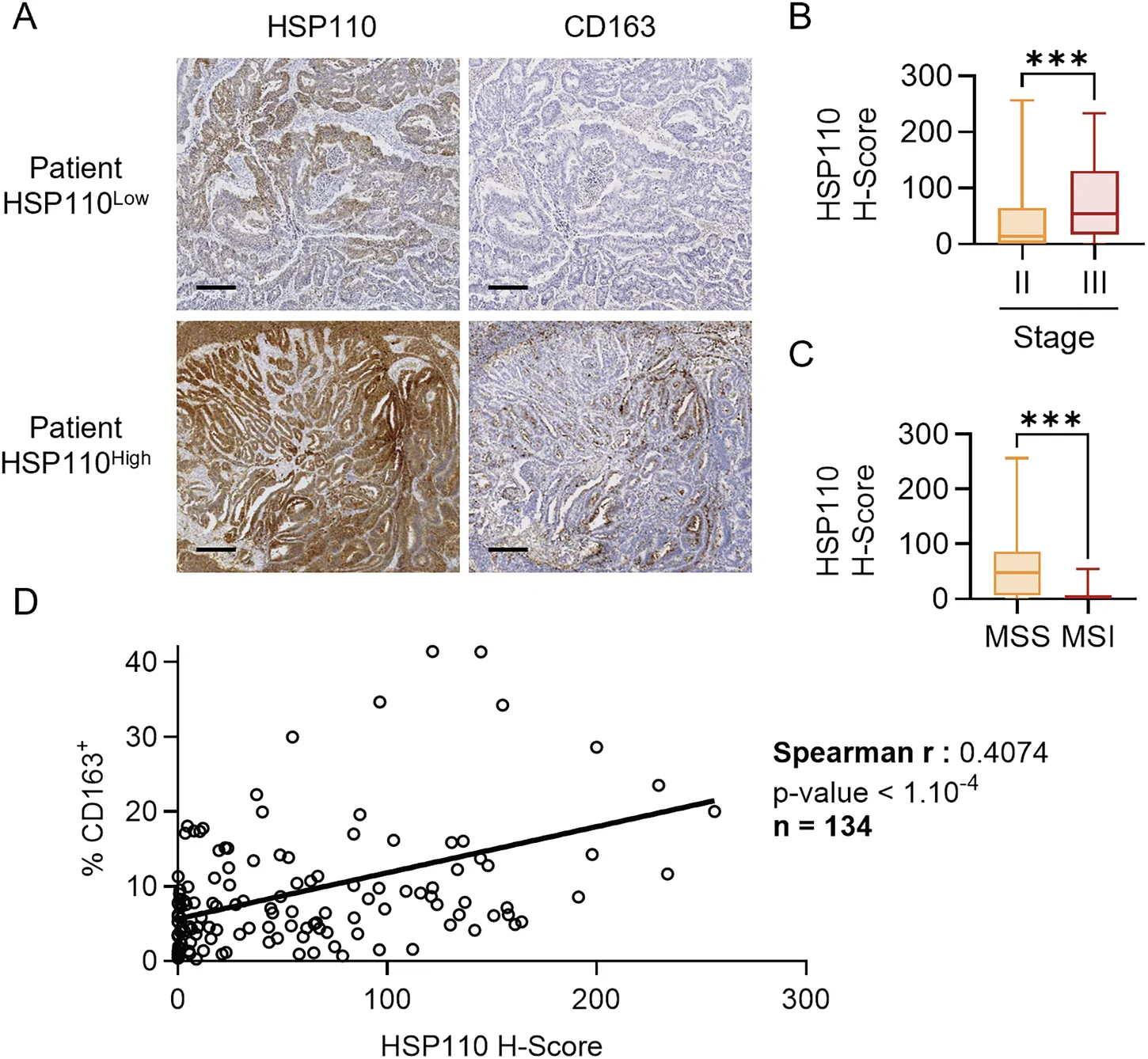
HSP110 correlated with the M2-like TAM functional marker CD163 in human biopsies from the PRODIGE-13 colorectal cancer cohort (134 patients). **A** Representative HSP110 and CD163 images observed after immunostaining (brown) of the tumor biopsies. Scale bar = 250 µm. Boxplots with median, quartiles, and Tukey’s whiskers displaying HSP110 H-score evaluation in stage 2 versus stage 3 patients (**B**), and MSI versus MSS patients (**C**). Mann–Whitney test: ***p < 0.001. **D** Correlation was observed for the expression of HSP110 and CD163. The Spearman correlation coefficient is indicated.

To substantiate the clinical relevance of HSP110 effect on macrophages, we analyzed their association in tumor biopsies from CRC stage 2 and 3 patients (*n* = 134) from the randomized, prospective multicenter study PRODIGE-13 (NCT00995202, sponsored by Fédération Francophone de Cancérologie Digestive). An H-score strategy was used to quantify HSP110 within the tumor biopsies to limit a potential bias due to tissue staining heterogeneity. First, and in accord with our previous results in this cohort demonstrating that HSP110 associated with bad prognosis [4], we found that the HSP110 H-score was significantly higher in stage 3 tumors compared to stage 2 (Median: 54.86 (IQR: 113.73) vs. 14.01 (IQR: 64.17); *p*-value: 3.10^−4^). Further and also as expected, microsatellite instable (MSI) tumors that all bear the inactivating mutation in the HSP110 gene and had a better prognosis than the microsatellite stable (MSS) [3], had a much lower HSP110 H-score than the MSS tumors (Median: 1.309 (IQR: 7.17) vs. 47.57 (IQR: 79.73), *p*-value: 3.10^−4^) (Fig. 6A–C). To analyze the association between HSP110 expression and anti-inflammatory macrophages infiltrating the tumor we used CD163, a reliable M2-like biomarker in robotized unbiased immunohistochemistry protocols. As shown in Fig. 6A, D HSP110 H-score correlated with CD163 expression, with a Spearman correlation coefficient of 0.4.

## Discussion

The emergence of HSP-targeting drugs as potential anticancer agents is based on the idea that HSPs may function similarly to oncogenes, aiding the adaptation of tumor cells to the hostile microenvironments [6, 21]. Among the various HSPs studied in cancer research, HSP110 has been relatively overlooked. A significant breakthrough occurred when HSP110 was found to be mutated in ~15% of CRC and gastric cancer patients, with this loss-of-function mutation associated with an excellent prognosis, particularly in terms of response to therapy [3, 4, 22]. This inactivated chaperone, the only HSP ever found mutated in a tumor, underscores the potential clinical significance of targeting HSP110 in cancer therapy.

In this study, we used biopsies issued from the PRODIGE-13 trial (*n* = 134 CRC patients) to demonstrate that the association of HSP110 with poor prognosis that we already reported [4] correlated with a higher number of anti-inflammatory macrophages within the tumor microenvironment. This finding aligns with the reported role of HSP110 in macrophage function [8, 15].

Seeking inhibitors of HSP110 that could be used in the clinic, we investigated the antitumor properties of a second-generation HSP110 inhibitor, C7 [16]. We first established here the superior anti-tumor effectiveness of this 2.0 inhibitor compared to the first-in-class hit, C33 [15]. C7 demonstrated enhanced efficacy in inhibiting HSP110 thereby impacting cancer cells growth and inflammatory macrophage polarization in vitro and in vivo. Further, our results showed that C7 upregulated PD-L1 expression in both macrophages and tumor cells.

ICIs, and notably the antiPD-L1/PD-1, are revolutionizing the treatment of patients with advanced/metastatic cancers. While these therapies have markedly improved patient survival, the challenge persists as many cancer patients remain unresponsive (about 40% of all cancers). Although the mechanisms determining responsiveness or resistance to ICIs are still poorly understood, emerging evidence suggests that cells of the innate immune system, such as macrophages infiltrating the tumor, may play a crucial role [19]. This, coupled with the fact that C7, like many chemical anticancer drugs, induced PD-L1 expression, prompted us to examine its effect in combination with an anti-PD-L1 therapy.

In this work, we demonstrated in two syngeneic mouse models of CRC - one resistant to anti-PD-L1 therapies (CT26) and the other sensitive (MC38) – the interest of associating C7 with the ICI. The potentiation effect observed resulted from the combined impact of 1) C7 blocking HSP110-induced STAT3 phosphorylation/tumor cell proliferation, inhibition of apoptosis (caspase 3), as well as its effect on anti-inflammatory macrophages; 2) anti-PD-L1 increasing the number and activity of cytotoxic T cells in the tumor microenvironment. The synergistic effect in the PD-L1-resistant model may result from C7 direct effect on macrophages thereby inducing the reshaping of the tumor microenvironment from a suppressive state to a pro-inflammatory state. This shift can directly impact adaptive immunity, allowing CD8 T cells to remain active and responsive. The efficacy of this response is further amplified by PD-L1 blockade, enabling CD8 T cells to mount an effective and robust response against the tumor.

Our data show that C7 increases PD-L1 expression in tumor cells and TAM but does not directly inhibit CD8⁺ T cell function (Fig. 5D). Rather, C7 reprograms macrophages toward a pro-inflammatory phenotype that supports CD8⁺ T cell proliferation and IFNγ production (Fig. 5B, C). Although in the MC38 model the reduction in tumor growth induced by C7 monotherapy is not as important (and therefore not always statistically significant) as that observed in the CT-26 model, the most robust antitumor effects in both models were always observed with C7 and anti-PD-L1 combination, consistent with a mechanism whereby C7 reprograms macrophages and PD-L1 blockade counteracts adaptive resistance, leading to enhanced CD8⁺ T cell–mediated cytotoxicity.

In this study, treatments in the tumor-bearing mice did not extend beyond 3 weeks, since the experimental endpoint was defined by the analysis of TILs to capture early immune changes. As a result, the long-term therapeutic effect was not assessed. Future studies will be needed to evaluate treatment durability through extended survival protocols, tumor rechallenge experiments, and to assess immune memory responses.

It should be mentioned that we also demonstrated that the first-in-class compound C33, though less potent than C7, also sensitizes CT26 tumors to anti-PD-L1 therapy. In line with these results, it has been recently reported using the same CT26 model that blockage of CD47, a molecule involved in the phagocytosis of tumor cells by the macrophage, increased the therapeutic effect of PD-L1 and FOLFOX regiments [23].

The optimization of anticancer immunotherapies is booming, particularly those concerning anti PD-L1/PD-1. However, few involve the use of molecules targeting directly macrophages/myeloid cells with the objective of decreasing the tumor-permissive and immunosuppressive characteristics of the TAM. Several of them are inhibitors of the colony-stimulating factor-1 receptor (CSF1R), like the FDA-approved PLX3397, or agonistes of the tumor necrosis factor receptor superfamily member CD40. For both of them an encouraging phase 1 trial has already been performed [24–26], suggesting the clinical feasibility of this strategy.

Altogether, our findings here highlight the potential of HSP110 inhibitors as a valuable approach to improve the efficacy of anti-PD-L1. This strategy offers a promising avenue for future translation to patients who may not respond adequately to the ICI alone, holding promise for improving CRC treatment outcomes and expanding the benefits of ICIs to a broader spectrum of patients.

## Supplementary information

Supplementary information

## Data Availability

Data are available on reasonable request. All data relevant to the study are included in the article or uploaded as online supplementary information.
