# Supplementary material for: Combining PD-L1 blockade with a second-generation HSP110 inhibitor enhances antitumor immunity in colorectal cancer
Source: Cell Death Dis. 2026 May 19;17(1):753. doi: 10.1038/s41419-026-08826-7 (PMC13503828; doi:10.1038/s41419-026-08826-7)
Supplement: Supplementary file 1 [file 41419_2026_8826_MOESM1_ESM.pdf]

SUPPLEMENTARY INFORMATION

Combining PD-L1 blockade with a second-generation HSP110 inhibitor enhances antitumor immunity in colorectal cancer

María Jimena Abrey-Recalde[1,#], Flavie Mialhe[1,#], Thi Khanh Le[1], Abdelmnim Radoua[1], Lisa Lagorgette[1], Rim Belkaid[1], Adiilah Mamode Cassim[1], Daniel Gonzalez[2], Elise Jacquin[1], Jessica Gobbo[3], Frédéric Bibeau[2,4], Côme Lepage[5], Li Xu[6], Xingrui He[6], Valentin Derangère[7], Frédéric Lirussi[1,8], Gaëtan Jego[1], Xiang-Yang Ye[6,‡], François Hermetet[9,‡,#], Carmen Garrido[1,3,#]

[1] *Université Bourgogne Europe, INSERM, CTM UMR 1231, HSP-Pathies Team, équipe labellisée « Ligue Nationale Contre le Cancer », 21000 Dijon, France*
[2] *Université Bourgogne Europe, Fédération Francophone de Cancérologie Digestive (FFCD), INSERM, CTM UMR 1231, EPICAD Team, 21000 Dijon, France*
[3] *Université Bourgogne Europe, Centre de Lutte Contre le Cancer G.-F. Leclerc, Unicancer, INSERM, CTM UMR 1231, HSP-Pathies Team, équipe labellisée « Ligue Nationale Contre le Cancer », 21000 Dijon, France*
[4] *Université Marie et Louis Pasteur, CHU Besançon, Department of Pathology, 25000 Besançon, France*
[5] *Université Bourgogne Europe, CHU Dijon Bourgogne, Department of Digestive Oncology, Fédération Francophone de Cancérologie Digestive (FFCD), INSERM, CTM UMR 1231, EPICAD Team, 21000 Dijon, France*
[6] *School of Pharmacy, Hangzhou Normal University, Hangzhou, Zhejiang, 311121, China*
[7] *Université Bourgogne Europe, Centre de Lutte Contre le Cancer G.-F. Leclerc, Unicancer, INSERM, CTM UMR 1231, TIRECs Team, 21000 Dijon, France*
[8] *Université Marie et Louis Pasteur, CHU Besançon, Department of Clinical Pharmacology and Toxicology, PACE Platform, Metabolomics Unit, 25000 Besançon, France*
[9] *Université Bourgogne Europe, INSERM, CTM UMR 1231, TIRECs Team, 21000 Dijon, France*

[#] Equal contribution
[‡] Corresponding authors

François Hermetet
Université Bourgogne Europe, INSERM, CTM UMR 1231, TIRECs Team
Faculty of Medicine and Pharmacy
21000 Dijon, France
Email: Francois.Hermetet@ube.fr / Francois.Hermetet@inserm.fr

Xiang-Yang Ye
School of Pharmacy, Hangzhou Normal University, Hangzhou,
Zhejiang, 311121, China
Email: xyye@hznu.edu.cn

All authors declare no conflicts of interest.

SUPPLEMENTARY FIGURES

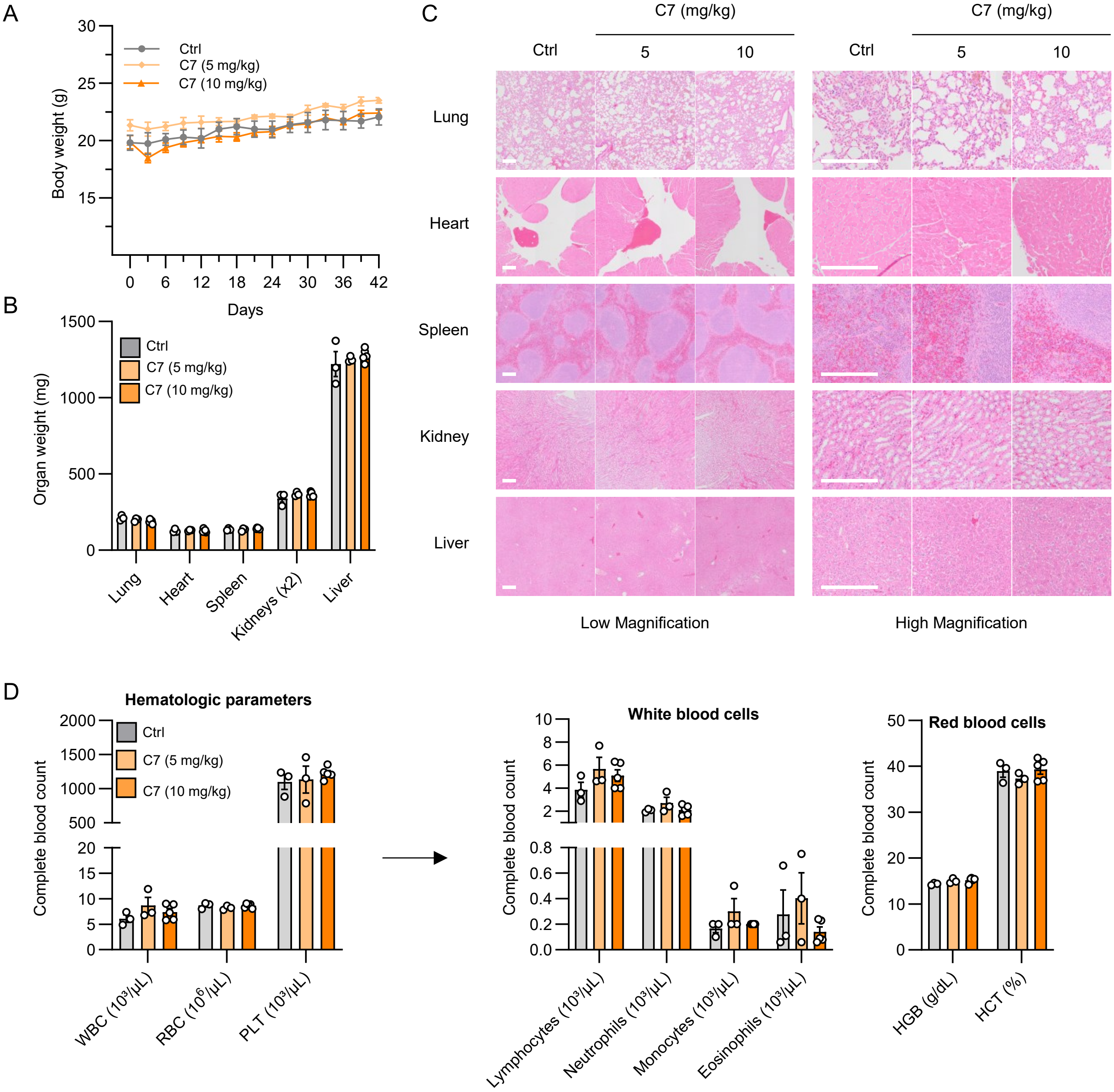

Figure S1. C7 safety evaluation in BALB/c mice (6-week study). (A) Body weight monitoring of BALB/c mice treated with vehicle (Ctrl) or C7 compound (5 or 10 mg/kg) over a 6-week period, showing no significant differences among groups. **(B)** Organ weight. **(C)** Representative hematoxylin and eosin (H&E)-stained sections of major organs (lung, heart, spleen, kidney, and liver) from control and C7-treated mice, showing no observable histopathological alterations at either low or high magnification. Scale bar: 200 µm. **(D)** Blood cell counts, including total white and red blood cell counts, as well as differential leukocyte populations, demonstrating no significant hematological abnormalities following C7 administration. HCT: Hematocrit, HGB: Hemoglobin, PLT: Platelets, RBC: Red blood cells, WBC: White blood cells. Data are presented as mean ± SEM (n = 3-5 per group).

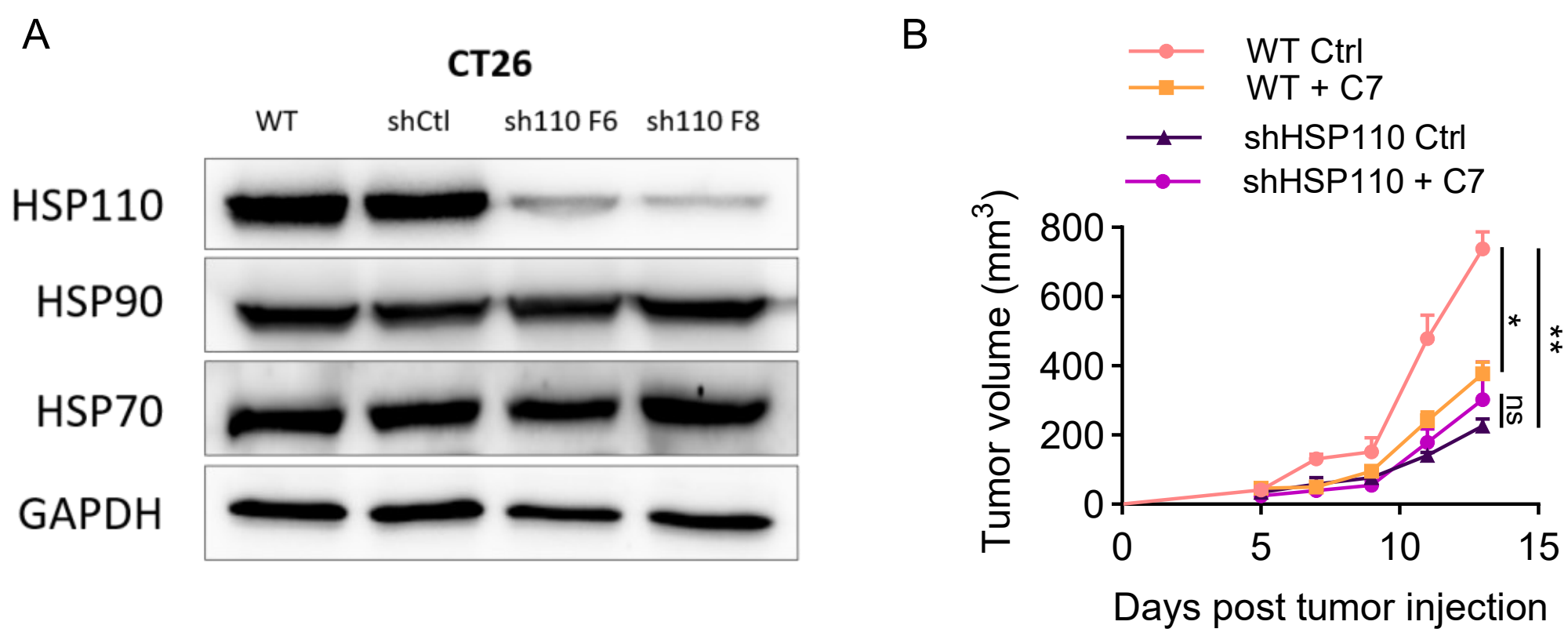

Figure S2. HSP110 knockdown inhibits C7 anti-tumor effect. (A) A shRNA lentiviral approach was used to stably knockdown HSP110 in the CT26 cell line. HSP110, HSP90, HSP70 expression was assayed by western blot. **(B)** Tumor volume monitoring of CT26 WT or shHSP110 in BALB/c mice control and treated with C7 (5 mg/kg). Animals were treated (i.p.) 3 times per week. Data are depicted as means ± SEM (n ≥ 3). Two-way ANOVA: *p < 0.05; **p < 0.01; ns, not significant.

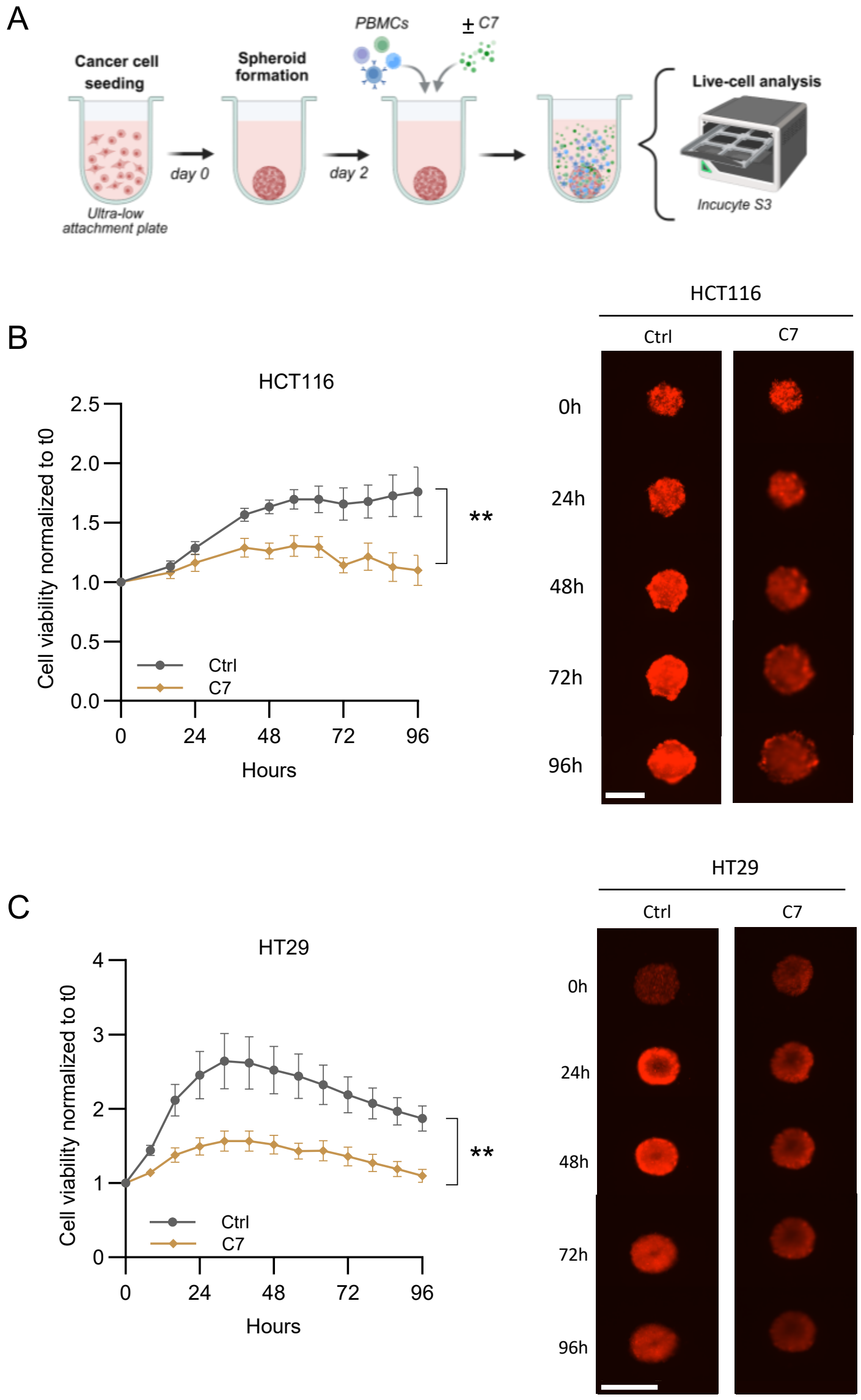

Figure S3. C7 treatment inhibits HCT 116 and HT-29 spheroids growth in 3D co-cultures. (A) Schema depicting the protocol used. Briefly, fluorescent HCT 116 and HT-29 spheroids (mKate2$^+$) were generated in ultra-low attachment plates and co-cultured with peripheral blood mononuclear cells (PBMCs) from healthy donors at a 1:1 ratio. Cells were treated with HSP110 inhibitor C7 (40 µM) or control vehicle. Spheroid fluorescence was monitored for 96 h using IncuCyte live-cell imaging. **(B, C)** Quantification of normalized spheroid fluorescence over time and representative images showing spheroid size and integrity at the indicated time points for HCT 116 **(B)** and HT-29 **(C)**. Scale bar: 800 µm. C7 treatment significantly inhibited spheroid growth in both cell lines co-cultured with PBMCs. Data are expressed as mean ± SEM (n = 4) from 2 independent donors. Statistical significance was determined by two-way ANOVA (**p < 0.01).

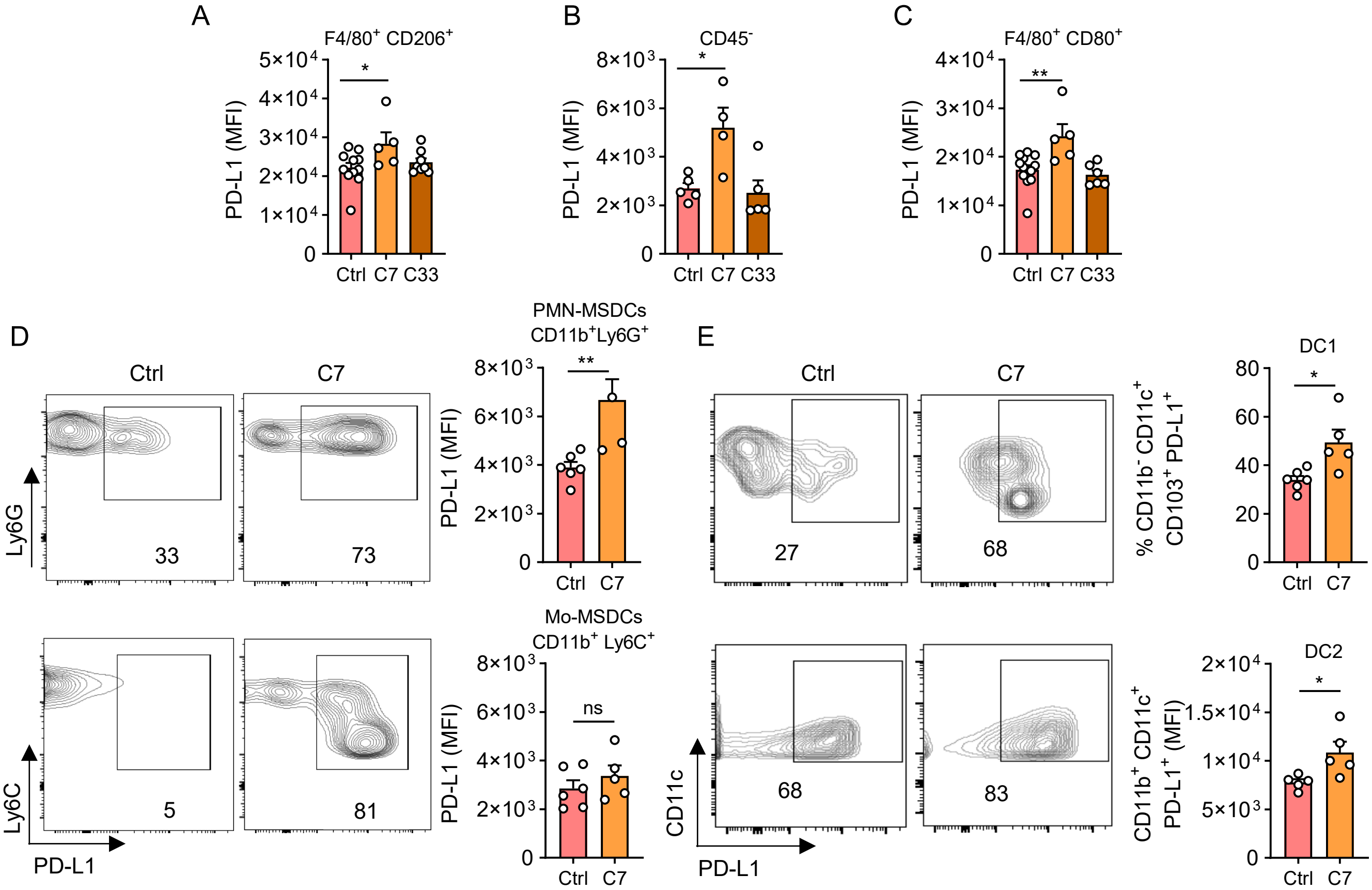

Figure S4. C7 induces PD-L1 expression in cancer cells, tumor associated macrophages (TAMs), tumor infiltrating dendritic cells (DC) and myeloid-derived suppressor cells (MDSCs). (A) Expression of PD-L1 by M2-like macrophages (F4/80$^+$CD206$^+$) in CT26 tumors from control BALB/c mice and treated with C7 or C33 was determined by flow cytometry. **(B)** Expression of PD-L1 in tumor cells (CD45$^-$) from control mice or treated with compound C7 or C33. **(C)** Expression of PD-L1 by M1-like macrophages (F4/80$^+$CD80$^+$) in tumors from mice control and treated with C7 or C33. **(D)** MDSCs and their PD-L1 expression were evaluated in tumors from control or C7-treated mice. Results are expressed as mean (± SEM, n = 5) of percentage Ly6C^{high} LY6G$^-$ for Mo-MDSCs and LY6C^{lo}LY6G$^+$ for PMN-MDSCs. **(E)** Dendritic cells (DCs) and their PD-L1 expression were evaluated in tumors of control or C7-treated mice. Results are expressed as mean (± SEM, n = 5) of percentage CD11b$^+$ CD11c$^+$ for DCs type 2 and CD11b$^-$ CD11c$^+$ CD103$^+$ for DCs type 1. PD-L1 expression is represented as mean (± SEM, n ≥ 5). of percentage or fluorescence intensity. One-way ANOVA **(A-C)** and unpaired t-test **(D, E)**: $*p < 0.05$; $**p < 0.01$; ns: not significant.

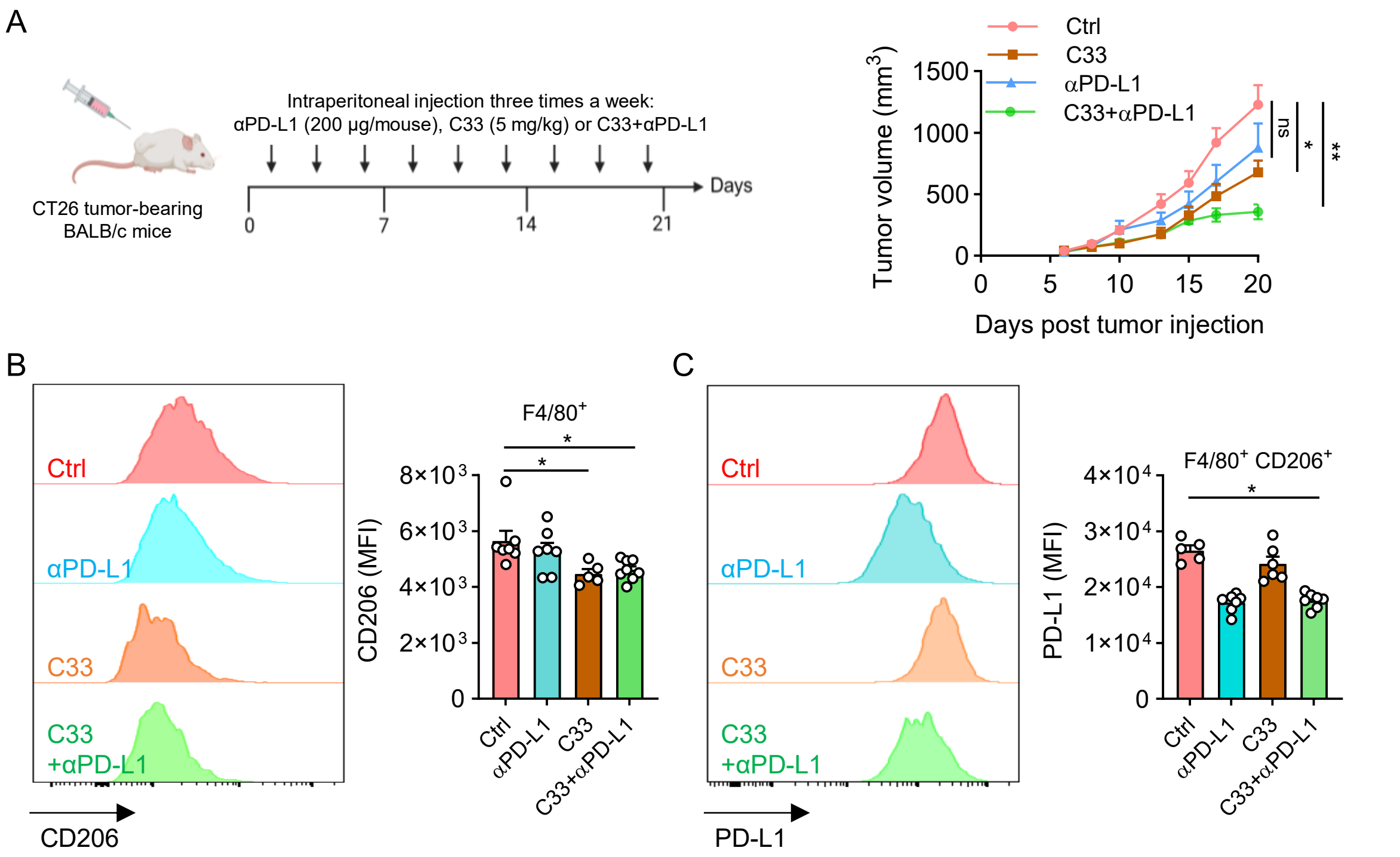

Figure S5. Combinatory effect of compound C33 and anti-PD-L1 in vivo. (A) CT26 tumor volume in BALB/c mice control or treated with C33, anti-PD-L1 or the combination. Animals were treated (i.p.) 3 times per week. Mean volume (± SEM) is represented ($n \geq 8$). Two-way ANOVA: *$p < 0.05$; **$p < 0.01$; ns: non-significant. **(B)** Expression of CD206 by TAMs from tumors of control and treated mice was evaluated by flow cytometry. Represented as the mean (± SEM, $n = 6$) of CD206 fluorescence intensity in F4/80^{+} cells. Kruskal-Wallis test: *$p < 0.05$. **(C)** PD-L1 expression in M2-like macrophages associated to tumors from control, C33, anti-PD-L1 or C33+antiPD-L1-treated mice. Mean (± SEM, $n \geq 5$) of PD-L1 fluorescence intensity is represented. One-way ANOVA: ****$p < 0.0001$.

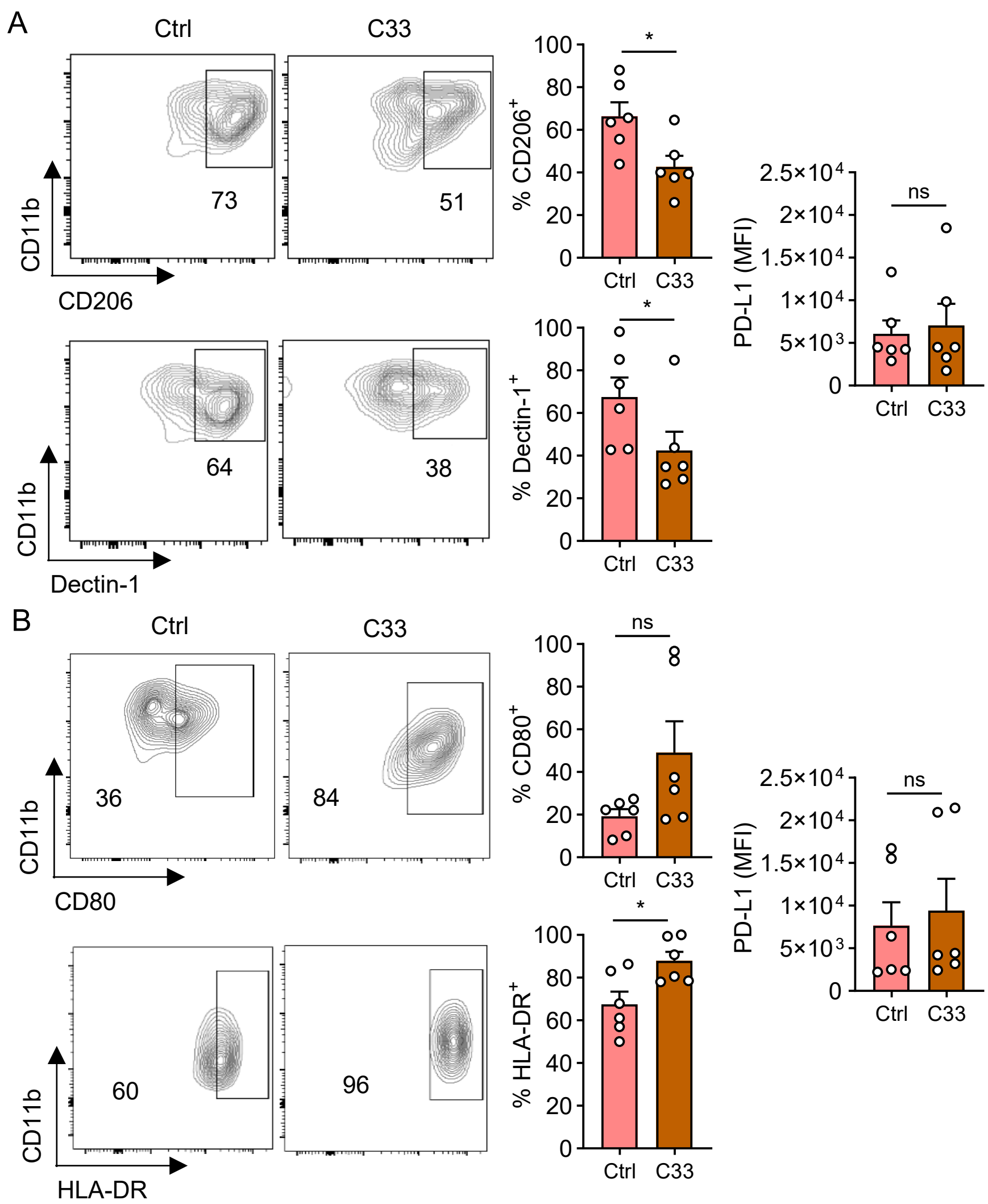

Figure S6. C33 induces macrophage polarization towards an M1 phenotype. Differentiated M2-like **(A)** or M1-like **(B)** macrophages in the presence or not of C33 were assessed for the expression of CD206, Dectin-1, HLA-DR, CD80, and PD-L1 by flow cytometry. Results are expressed as mean (± SEM, n = 6) of percentage of positive cells or mean fluorescence intensity. Paired t-test and Wilcoxon test: *p < 0.05; ns, not significant.

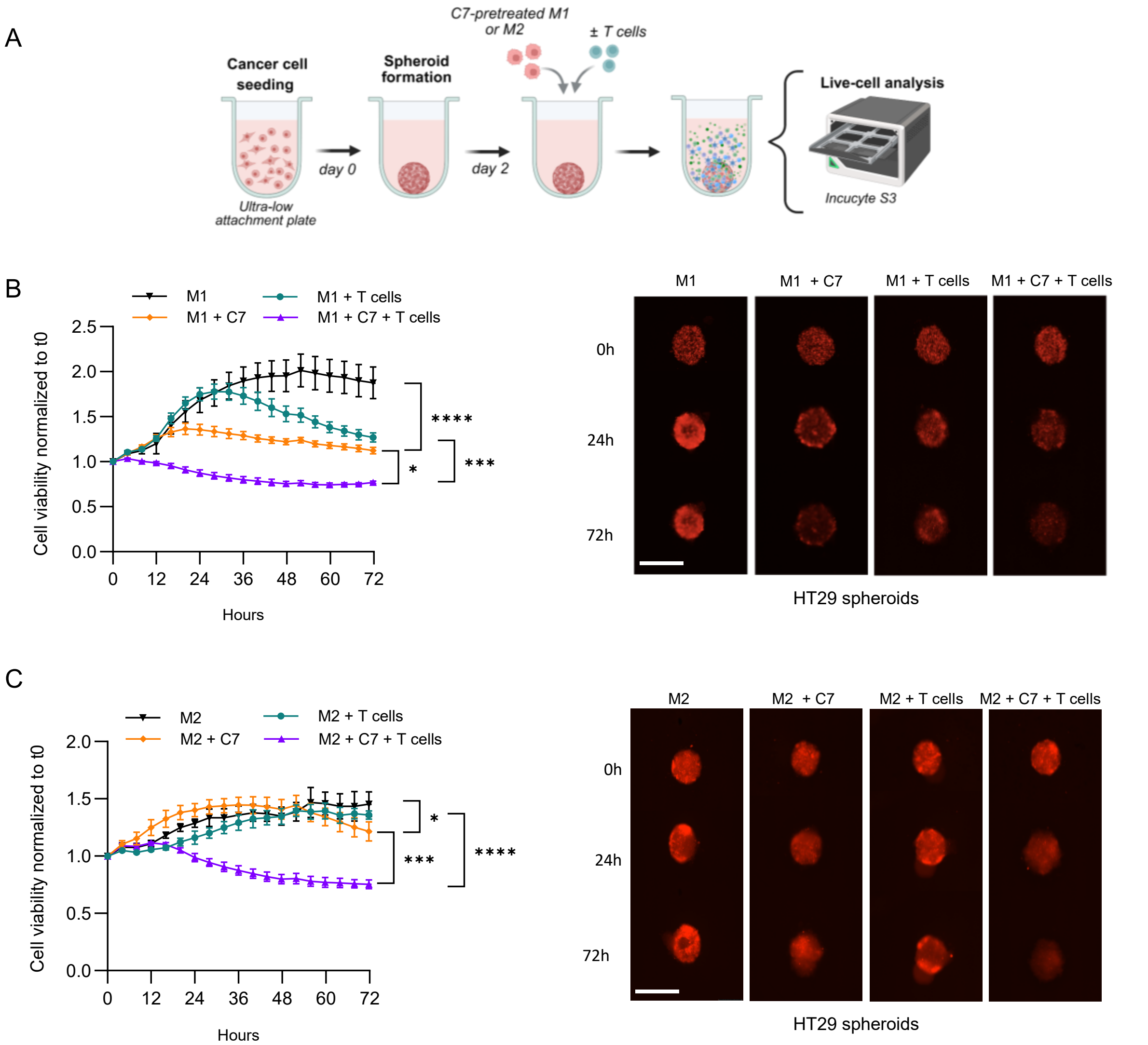

Figure S7. C7-conditioned macrophages inhibit HT-29 spheroid growth in 3D co-culture. (A) Schema depicting the protocol used. HT-29 spheroids, generated in ultra-low attachment plates, were co-cultured with PBMC-derived M1- or M2-like macrophages (previously pretreated or not with 40 µM C7) with or without autologous activated T cells. HT-29 cells and macrophages were seeded at a 2:1 ratio; T cells were added at a 1:1 ratio. Spheroid growth was monitored by live-cell imaging (IncuCyte S3). **(B)** Quantification and representative images of HT-29 spheroids co-cultured with M1-like macrophages at the indicated times. **(C)** Quantification and representative images of HT-29 spheroids co-cultured with M2-like macrophages. C7-conditioned M2 macrophages decreased spheroid fluorescence, consistent with pro-inflammatory repolarization and enhanced macrophage–T cell interaction. Scale bar: 800 µm. Data are expressed as mean ± SEM (n = 3); Statistical significance was determined by two-way ANOVA (*p < 0.01; **p < 0.001; ***p < 0.001; ****p < 0.0001; ns, not significant).

Full Western Blot Images

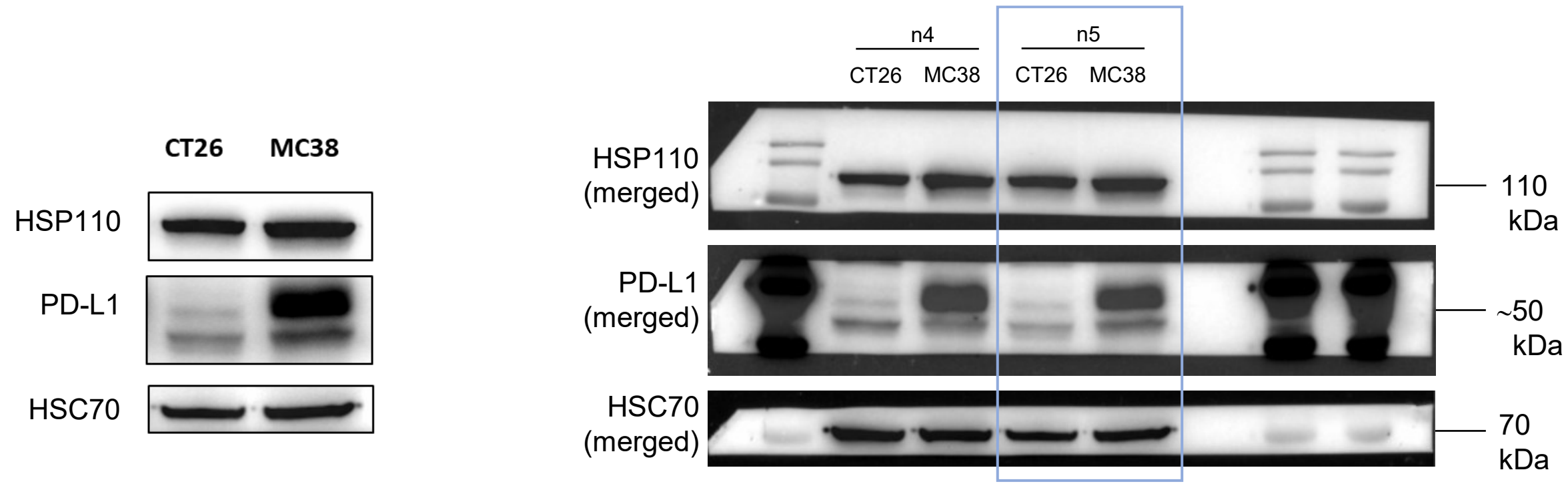

Uncropped merged Western blot (Figure 2B)

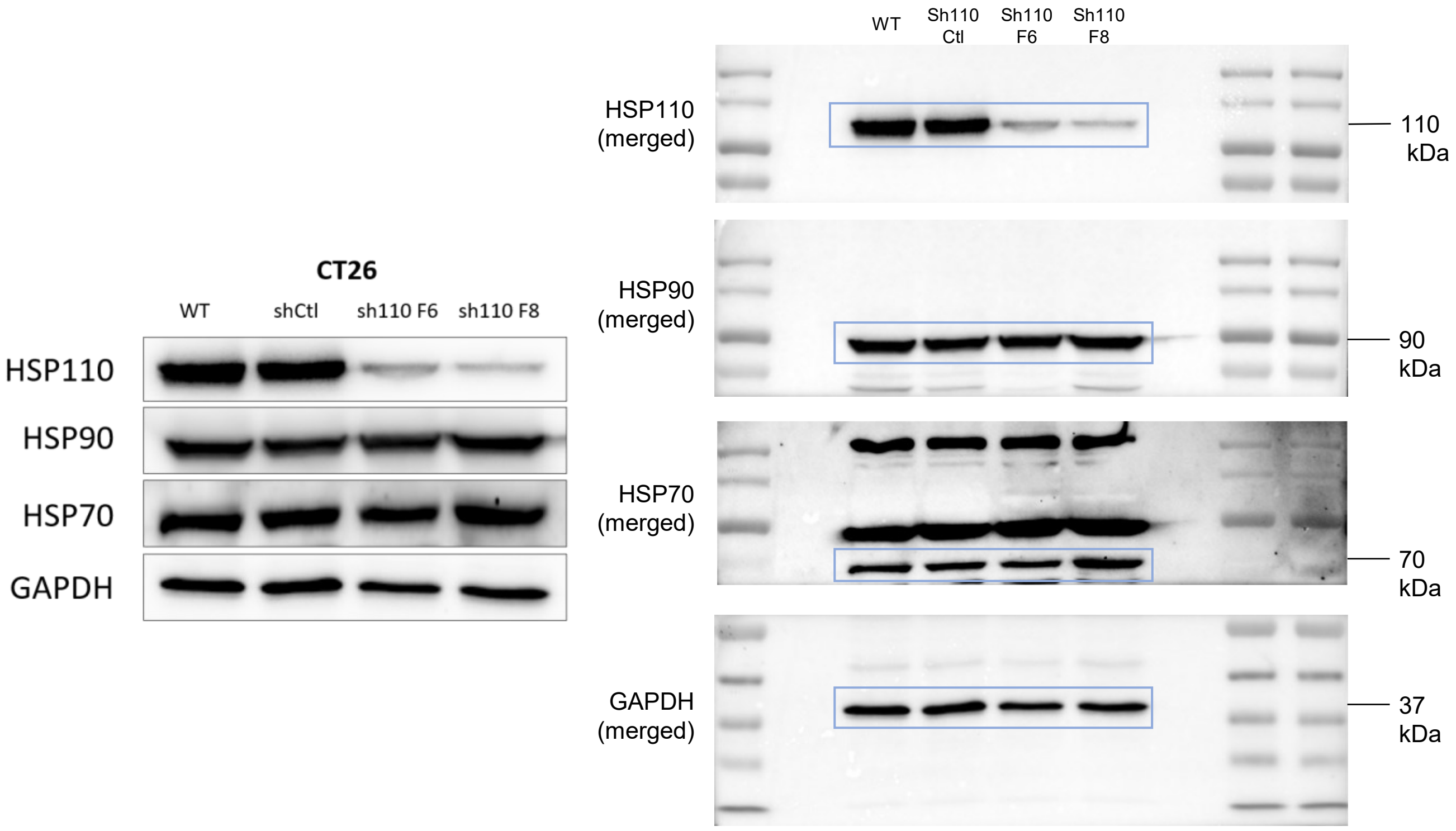

Uncropped merged Western blot (Figure S2A)
